# Palladium-catalyzed selective oxidation of ethane to acetate acid

**DOI:** 10.1093/nsr/nwaf355

**Published:** 2025-08-23

**Authors:** Ting Li, Baotong Huang, Weijie Li, Xin Deng, Yuchao Chai, Guangjun Wu, Xiufang Xu, Landong Li

**Affiliations:** Key Laboratory of Advanced Energy Materials Chemistry of Ministry of Education, College of Chemistry, Nankai University, Tianjin 300071, China; Key Laboratory of Advanced Energy Materials Chemistry of Ministry of Education, College of Chemistry, Nankai University, Tianjin 300071, China; Key Laboratory of Advanced Energy Materials Chemistry of Ministry of Education, College of Chemistry, Nankai University, Tianjin 300071, China; Key Laboratory of Advanced Energy Materials Chemistry of Ministry of Education, College of Chemistry, Nankai University, Tianjin 300071, China; Key Laboratory of Advanced Energy Materials Chemistry of Ministry of Education, College of Chemistry, Nankai University, Tianjin 300071, China; Key Laboratory of Advanced Energy Materials Chemistry of Ministry of Education, College of Chemistry, Nankai University, Tianjin 300071, China; Key Laboratory of Advanced Energy Materials Chemistry of Ministry of Education, College of Chemistry, Nankai University, Tianjin 300071, China; Key Laboratory of Advanced Energy Materials Chemistry of Ministry of Education, College of Chemistry, Nankai University, Tianjin 300071, China; Frontiers Science Center for New Organic Matter, Nankai University, Tianjin 300071, China

**Keywords:** C–H bond activation, homogeneous catalysis, palladium, ethane oxidation, acetic acid

## Abstract

The selective oxidation of ethane to acetate acid represents a promising route for ethane conversion, which is currently challenged by the high C–H bonding energy in ethane molecules and the incidental side reactions under employed conditions. We demonstrate herein that a simple PdCl_2_ catalyst can efficiently catalyze the selective oxidation of ethane to acetate acid in the reaction system of C_2_H_6_-O_2_-CO-H_2_O. The impacts of reaction parameters on ethane oxidation are systematically investigated. Kinetic and spectroscopic analyses reveal the reaction pathway starting from a water-gas-shift reaction, followed by *in-situ* hydroxyl radical and H_2_O_2_ formation for efficient ethane oxidation. In the refined reaction system with the addition of sulfuric acid, high ethane conversion of 15.7% and high acetate acid selectivity of 92.1% can be achieved at the same time, offering a state-of-the-art acetate acid space-time-yield of 372.7 mol_CH3COOH_ mol_Pd_^−1^ h^−1^ with great potential for practical applications. Finally, the reaction pathway and mechanism of ethane oxidation are interpreted by theoretical calculations to shed light on the rational design of reaction systems for natural gas upgrading.

## Teaser

A simple PdCl2 catalyst can efficiently catalyze the selective oxidation of ethane to acetic acid using O_2_ and CO, presenting a potential strategy for natural gas upgrading under mild conditions.

## INTRODUCTION

With the increasing demand for energy and high-value chemicals, the utilization of natural gas has attracted widespread attention. Current research predominantly focuses on the upgrading of methane, while the efficient conversion of ethane, the second most abundant component in shale gas (1%–10%), also holds substantial significance [1]. Ethane is primarily transformed to ethylene through steam cracking, which is subsequently employed in the production of polyethylene or oxygenates such as acetic acid, ethylene oxide, acetaldehyde, vinyl chloride, and ethanol [2]. Among these oxygenates, acetic acid is the precursor for the production of vinyl acetate and acetate fibers, playing an important role in the modern chemical industry. Currently, acetic acid is mainly produced from the carbonylation of methanol while it can also be produced from the selective oxidation of acetaldehyde or even ethane.

The high energy barrier for C–H bond cleavage (423.3 kJ mol^−1^) during ethane oxidation generally necessitates elevated reaction temperatures, which in turn promotes the side reactions from C–C bond cleavage and leads to catalyst deactivation under harsh conditions, ultimately leading to low ethane conversion efficiency [3,4]. The oxidation of ethane to acetic acid is a complex reaction involving multiple steps, and catalysts play a crucial role in determining the reaction pathway and product selectivity. Thereupon, exploring a catalyst that can facilitate the ethane oxidation while minimizing other undesired side reactions is of paramount importance. Previous studies have demonstrated that supported noble metal catalysts are active for the selective oxidation of ethane. For example, the complex Mo-V-Ln-Nb-Pd-X catalysts developed by SABIC could achieve 85% acetic acid selectivity in ethane oxidation by O_2_ at 280°C and 1.38 MPa, while the low ethane conversion (∼10%) and space-time-yield (STY) limit its industrial viability [5]. Lin and Sen demonstrated that Pd/C and Pt/C could catalyze the conversion of ethane to acetic acid in the presence of CO, O_2_, and an acidic medium [6]. V-containing heteropolyacids were employed in the direct transformation of light alkanes into the corresponding carboxylic acids in the system of K_2_S_2_O_8_/CF_3_COOH [7]. More recently, Fe- and Rh-based zeolites exhibited impressive performance in H_2_O_2_-mediated oxidation processes to produce high amounts of acetic acid and formic acid, and Ir-based clusters showed high efficiency in ethane oxidation to oxygenates by O_2_ with the assistance of CO [8–12]. Interestingly, acetic acid could be also produced from methane through sequential C–H bond activation and CO insertion over Rh-based catalysts [13–15]. However, the STY of acetic acid in the reaction system is generally very low and limited by the C–H bond activation step. Recent studies have demonstrated breakthroughs in the lattice oxygen-mediated oxidative conversion of ethane under mild conditions with the participation of light [16–18]. Pd catalysts have also shown significant potential in the oxidation of light alkanes towards carboxylic acids in a strong acidic medium [19–21] or (CF_3_CO)_2_O [22].

In this study, we demonstrate that simple ionic Pd(II) species can efficiently catalyze ethane oxidation to C_2_ oxygenates like acetic acid in the reaction system of C_2_H_6_-O_2_-CO-H_2_O. The state-of-the-art performance, including ethane conversion, acetic acid selectivity, and STY can be achieved at the same time through the optimization of reaction parameters. Electron paramagnetic resonance (EPR) analyses combined with titration experiments reveal the *in-situ* formation of hydroxyl radicals (**·**OH) as well as H_2_O_2_ and their functionalities as reactive oxygen species for ethane oxidation. The carbon and oxygen sources of oxygenate products are traced by isotope-labelling mass spectroscopy (MS), gas chromatography mass spectrometry (GC-MS) and nuclear magnetic resonance (NMR). Finally, the ethane oxidation pathway in the complex reaction system of C_2_H_6_-O_2_-CO-H_2_O is clearly interpreted by density functional theory (DFT) calculations to shed light on the reaction design.

## RESULTS AND DISCUSSION

### Catalytic evaluation for ethane oxidation

**Figure 1.**
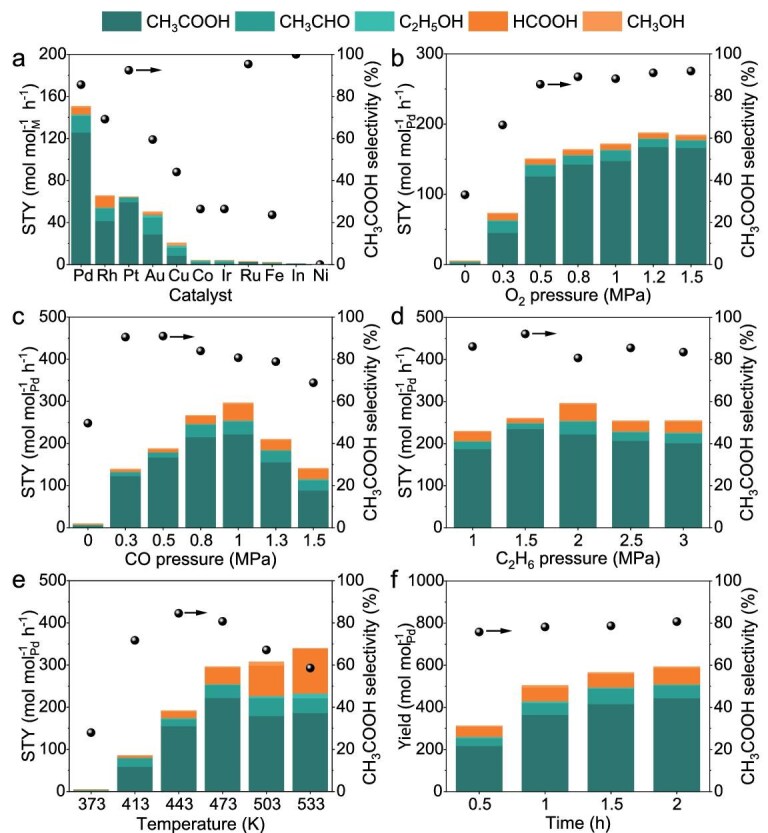
Performance in ethane selective oxidation (a) Ethane oxidation catalyzed by soluble metal ions. Reaction conditions: 2.9 μmol metal, 2.0 MPa C_2_H_6_, 0.5 MPa O_2_, 0.5 MPa CO, 10 mL H_2_O, 473 K, 2 h. (b) Impacts of O_2_ partial pressure on ethane oxidation over PdCl_2_ catalyst. Reaction conditions: 2.9 μmol Pd, 2.0 MPa C_2_H_6_, 0–1.5 MPa O_2_, 0.5 MPa CO, 10 mL H_2_O, 473 K, 2 h. (c) Impacts of CO partial pressure on ethane oxidation over PdCl_2_ catalyst. Reaction conditions: 2.9 μmol Pd, 2.0 MPa C_2_H_6_, 1.2 MPa O_2_, 0–1.5 MPa CO,10 mL H_2_O, 473 K, 2 h. (d) Impacts of C_2_H_6_ partial pressure on ethane oxidation over PdCl_2_ catalyst. Reaction conditions: 2.9 μmol Pd, 1.0–3.0 MPa C_2_H_6_, 1.2 MPa O_2_, 1 MPa CO, 10 mL H_2_O, 473 K, 2 h. (e) Temperature-dependent behaviors of ethane oxidation over PdCl_2_ catalyst. Reaction conditions: 2.9 μmol Pd, 2.0 MPa C_2_H_6_, 1.2 MPa O_2_, 1.0 MPa CO, 10 mL H_2_O, 373–533 K, 2 h. (f) Time-dependent behaviors of ethane oxidation over PdCl_2_ catalyst. Reaction conditions: 2.9 μmol Pd, 2.0 MPa C_2_H_6_, 1.2 MPa O_2_, 1.0 MPa CO, 10 mL H_2_O, 473 K, 0.5–2.0 h.

The catalytic performance of various soluble metal ions, including Pd, Rh, Au, Pt, Cu, Ir, Fe, Ru, In, Co, and Ni, was initially evaluated for ethane oxidation in the reaction system of C_2_H_6_-O_2_-CO-H_2_O (Fig. 1a and Figs S1–S6). Typically, Pd(II) ions showed the highest oxygenate STY of 150.7 mol mol_Pd_^−1^ h^−1^, with notable selectivity of 85.7% towards acetic acid. Comparative studies reveal that PdCl_2_ (containing equimolar HCl in terms of Cl^−^ to increase solubility) outperforms other Pd(II) catalysts with various anions (Fig. S7). The impact of catalyst dosage on ethane oxidation was investigated (Fig. S8), giving an optimized Pd dosage of 0.0002 mol_Pd_ mol_C2H6_^−1^, which is the most efficient utilization of Pd. In the complex reaction system involving ethane, CO, and O_2_, the impacts of reaction parameters on ethane oxidation were systematically investigated [23]. As shown in Fig. 1b, increasing O_2_ partial pressure to 0.5 MPa significantly enhanced both oxygenate STY and acetic acid selectivity while a further increase in O_2_ partial pressure from 0.5 to 1.5 MPa led to only modest improvement. Kinetic studies provide insights into this trend: as O_2_ partial pressure increases from 0.3 to 0.5 MPa, the reaction orders for oxygenate and acetic acid formation were determined to be 1.44 and 2.02, respectively, indicating the crucial role of O_2_ in acetic acid formation (Fig. S9). Beyond 0.5 MPa, the reaction orders for oxygenate and acetic acid formation were close to zero (0.21 and 0.27, respectively), demonstrating that acetic acid formation was no longer significantly influenced by higher O_2_ pressures [24]. The impact of CO partial pressure was examined, showing a volcano-shaped relationship with oxygenate STY (Fig. 1c). The positive reaction orders of 0.64 and 0.51 for oxygenate and acetic acid, respectively, suggest the beneficial role of CO at moderate pressures. However, the oxygenate STY declined with increasing CO pressure beyond 1.0 MPa, and the reaction orders for both oxygenate and acetic acid formation became negative (Fig. S10). These observations suggest that excessive CO might inhibit the reaction, likely due to the strong interaction between CO and Pd(II) ions and change in the active Pd sites thereof [24]. The ethane pressure showed mild impacts on the reaction of ethane oxidation and the maximal oxygenate STY was achieved at ethane pressure of 2.0 MPa (296.1 mol mol_Pd_^−1^ h^−1^, Fig. 1d).

The temperature-dependent behaviors of ethane oxidation over the PdCl_2_ catalyst were investigated. As shown in Fig. 1e, the acetic acid STY increased with rising temperature and reached the maximum at 473 K, offering an acetic acid STY of 222.3 mol mol_Pd_^−1^ h^−1^ and 80.7% acetic acid selectivity (10.7% ethane conversion after reaction for 2 h). Higher temperatures would promote cleavage of the C–C bond, leading to reduced C_2_ oxygenate selectivity [4]. The time-dependent behaviors were further investigated. As shown in Fig. 1f, the oxygenate yield increased with time-on-stream in the first 1.5 h and almost reached steady-state. Under optimized reaction conditions, namely C_2_H_6_/O_2_/CO partial pressure of 2.0/1.2/1.0 MPa at 473 K for 0.5 h, the PdCl_2_ solution could efficiently catalyze the oxidation of ethane, producing oxygenates with a surprisingly high STY of 625.2 mol mol_Pd_^−1^ h^−1^. The apparent activation energy for the reaction was calculated to be 89.8 kJ mol^−1^ (Fig. S11). Based on its excellent performance, the recycling property of the homogeneous PdCl_2_ catalyst system was assessed. As shown in Fig. S12, the oxygenate productivity significantly increased after each successive catalytic cycle, while the CH_3_COOH selectivity dropped gradually. This implied that the Pd centers remained active after the ethane oxidation reaction, while the slightly reduced performance could be related to the changed chemical equilibrium and the intensified side reaction of C–C bond cleavage with prolonged reaction time. For practical consideration, the homogeneous PdCl_2_ catalyst should be separated from the reaction system *via* distillation and subjected to the next reaction.

### Reaction pathway of ethane oxidation

**Figure 2.**
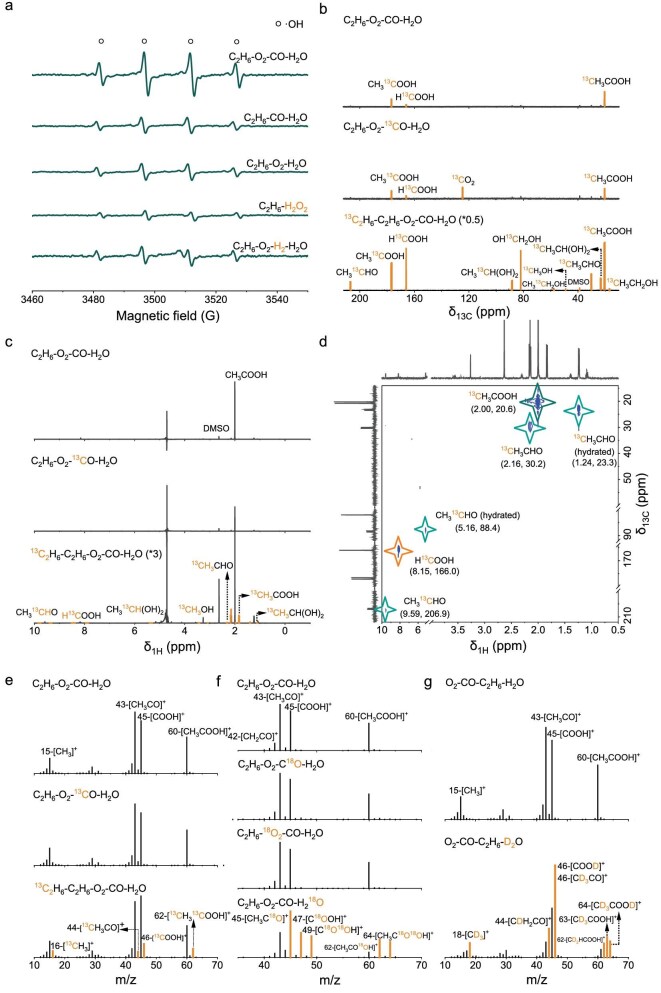
Reaction pathway of ethane selective oxidation. (a) EPR spectra of the reaction solution after ethane oxidation with different conditions. Reaction condition: (i) 2.9 μmol Pd, 1.2 MPa O_2_, 1 MPa CO, 2 MPa C_2_H_6_, 10 mL H_2_O, 473 K, 2 h; (ii) 2.9 μmol Pd, 1 MPa CO, 2 MPa C_2_H_6_, 10 mL H_2_O, 473 K, 2 h; (iii) 2.9 μmol Pd, 1.2 MPa O_2_, 2 MPa C_2_H_6_, 10 mL H_2_O, 473 K, 2 h; (iv) 2.9 μmol Pd, 2 MPa C_2_H_6_, 10 mL H_2_O_2_ (0.02 mol L^−1^), 473 K, 2 h; (v) 2.9 μmol Pd, 1.2 MPa O_2_, 1 MPa H_2_, 2 MPa C_2_H_6_, 10 mL H_2_O, 473 K, 2 h. (b) ^13^C NMR spectrum of isotopic ^13^C labelling experiments. (c) ^1^H NMR spectrum of ^13^C isotopic labelling experiments. (d) 2D ^1^H-^13^C HSQC spectra of ^13^C isotopic labelling experiments. (e) Acetic acid GC–MS spectra of ^13^C isotopic labelling experiments. (f) Acetic acid GC–MS spectra of ^18^O isotopic labelling experiments. (g) Acetic acid GC–MS spectra of D isotopic labelling experiment.

Given the exceptional ethane oxidation performance achieved with Pd(II) catalyst in the complex reaction system of C_2_H_6_-O_2_-CO-H_2_O, the reaction pathway was explored through a series of techniques including EPR, NMR, and GC-MS analyses. First, efficient ethane oxidation could only be achieved by the co-existence of CO and O_2_ (Fig. S13). A similar trend was found in the intensity of ·OH detected by EPR. As shown in Fig. 2a, significantly intensified ·OH signals were detected by the co-existence of CO and O_2_ in comparison with that employing CO or O_2_ alone. The catalytic data and EPR results demonstrate the necessity of CO-O_2_ co-existence for the formation of ·OH that acts as the reactive oxygen species for ethane oxidation. On this basis, the classical water-gas-shift reaction (WGSR, ${\mathrm{CO}} + {{\mathrm{H}}}_2{\mathrm{O\ }}{\rightarrow}{\mathrm{\ C}}{{\mathrm{O}}}_2 + {{\mathrm{H}}}_2$) could be proposed as the vital step for H_2_ generation, followed by the formation of hydrogen peroxide (H_2_O_2_) and ·OH upon reaction with O_2_ [25–28]. The reduced H_2_O_2_ amount by reacting with ethane further confirms the dynamic generation and consumption of H_2_O_2_ during the reaction process (Table S1). According to these results, two distinct reaction pathways of ethane oxidation to acetic acid can be proposed, namely ${{\mathrm{C}}}_2{{\mathrm{H}}}_6 + 1.5{{\mathrm{O}}}_2{\mathrm{\ }}{\rightarrow} {\mathrm{C}}{{\mathrm{H}}}_3{\mathrm{COOH}} + {\mathrm{\ }}{{\mathrm{H}}}_2{\mathrm{O}}$ (O_2_ pathway) and ${{\mathrm{C}}}_2{{\mathrm{H}}}_6 + 3{{\mathrm{O}}}_2 + 3{\mathrm{CO\ }}{\rightarrow}{\mathrm{C}}{{\mathrm{H}}}_3{\mathrm{COOH}} + 3{\mathrm{C}}{{\mathrm{O}}}_2 + {{\mathrm{H}}}_2{\mathrm{O}}$ (H_2_O_2_/·OH pathway). Although both reaction pathways are thermodynamically feasible, their stoichiometric ratios are quite different. The experimental ethane conversions were always lower than the theoretical maximum values in the H_2_O_2_/·OH pathway (Table S2), suggesting the dominant contribution of the H_2_O_2_/·OH pathway for acetic acid formation. In this case, the primary role of O_2_ in the reaction system is to react with H_2_ from WGSR for H_2_O_2_ and ·OH regeneration. For comparison, several ·OH-driven oxidation systems, including H_2_O_2_ and H_2_-O_2_, were investigated [25,29]. As shown in Fig. S13, the oxygenate STYs in the reaction systems employing H_2_O_2_ and H_2_-O_2_ were distinctly lower than those employing CO-O_2_, demonstrating the advantage of *in-situ* H_2_O_2_ and ·OH formation from the CO-O_2_-H_2_O system. Accordingly, the intensities of ·OH signals employing extraneous H_2_O_2_ and H_2_-O_2_ were distinctly lower than that employing CO-O_2_ (Fig. 2a).

To track the carbon source in the oxygenate products, isotope-labeling NMR analyses were performed. When ^13^C_2_H_6_ was employed as the reagent, the chemical shift signals at 20.6, 176.8, 30.2, 206.9, 23.3, 88.4, 16.9, 57.6, 166.0, and 49.1 ppm, corresponding to ^13^CH_3_COOH, CH_3_^13^COOH, ^13^CH_3_CHO, CH_3_^13^CHO, ^13^CH_3_CHO (hydrated), CH_3_^13^CHO (hydrated), ^13^CH_3_CH_2_OH, CH_3_^13^CH_2_OH, H^13^COOH, and ^13^CH_3_OH (detailed information shown in Table S3), were observed in the ^13^C NMR spectrum (Fig. 2b). In contrast, only the ^13^CO_2_ signal (124.7 ppm) emerged when employing ^13^CO as the reagent. These observations validate the WGSR and exclude the contribution of CO towards the liquid oxygenates. Signal splitting due to ^13^C–^13^C coupling was also evident in Fig. S14, reinforcing the incorporation of carbon in ethane into the oxygenate products. The satellite signals (two splitting signals) of ^13^CH_3_COOH (δ = 1.89, 2.05 ppm), ^13^CH_3_CHO (δ = 2.04, 2.20 ppm), CH_3_^13^CHO (δ = 9.44, 9.66 ppm), ^13^CH_3_CHO (hydrated) (δ = 1.13, 1.29 ppm), H^13^COOH (δ = 8.00, 8.27 ppm), and ^13^CH_3_OH (δ = 3.15, 3.33 ppm) were observed in the ^1^H NMR spectra (Fig. 2c and Fig. S15), while no isotope-labelled chemical shift signals could be detected when employing ^13^CO as the reagent, further confirming the sole carbon source from ethane. Two-dimensional heteronuclear single quantum correlation (2D-HSQC) provided direct evidence for the coupling of ^13^C and adjacent H of ^13^CH_3_COOH (2.00, 20.6), ^13^CH_3_CHO (2.16, 30.2), CH_3_^13^CHO (9. 59, 206.9), H^13^COOH (8.15, 166.0), ^13^CH_3_CHO (hydrated) (1.24, 23.3), CH_3_^13^CHO (hydrated) (5.16, 88.4) (Fig. 2d). Meanwhile, GC-MS analyses confirmed the presence of multiple ^13^C-labeled fragments, including ^13^CH_3_⁺ (*m/z* = 16), ^13^CH_3_CO⁺ (*m/z* = 44), ^13^COOH⁺ (*m/z* = 46), ^13^CH_3_^13^COOH (*m/z* = 62), etc., verifying ethane as the carbon source of the liquid oxygenates (Fig. 2e and Figs S16–S18). No ^13^C-labeled CH_3_COOH, CH_3_CHO, or HCOOH could be detected when employing ^13^CO as the reagent, excluding CO as the carbon source for the oxygenate products.

To track the oxygen source, isotope-labeling experiments employing C^18^O, ^18^O_2_, and H_2_^18^O were conducted. As shown in Fig. 2f, Figs S19 and S20, no ^18^O-labeled oxygenate products could be observed when employing C^18^O as the reagent. Ethanol displayed ^18^O-labeled signals (CH_2_^18^OH, *m/z* = 33; C_2_H_5_^18^OH, *m/z* = 48) when ^18^O_2_ was employed while no ^18^O-labeled signals were detected when H_2_^18^O was employed, indicating that O_2_ contributed to ethanol formation and the oxygen source of ethanol was O_2_ (Fig. S21). All aldehydes or carboxylic acids were ^18^O-labeled when H_2_^18^O was employed while no ^18^O-labeled products could be observed from the further oxidation of C_2_H_5_^18^OH. This might be explained by the fast exchange between aldehydes/acids and H_2_O. On this basis, the co-contribution of O_2_ and H_2_O to oxygenates could be confirmed although ^16^O-^18^O exchange occurred by mixing CH_3_COOH, CH_3_CHO, or HCOOH with H_2_^18^O under employed conditions (Fig. S22). It could be inferred that the oxygen source of ethanol and acetic acid was derived from O_2_ and H_2_O, respectively [23]. As for D-labeled experiments, the similar role of H_2_O could be confirmed by all products labeled with D (Fig. 2g, Figs S23 and S24) despite the H-D exchange in the hydroxyl group of aldehyde or carboxylic acids (Fig. S25).

The gas phase products were monitored by mass spectroscopy. As shown in Fig. S26, the signals of *m/z* = 2 and 44, corresponding to H_2_ and CO_2_, could be observed under employed conditions, further confirming the occurrence of the WGSR. The signals of ^13^CO_2_ (*m/z* = 45) and C^18^OO (*m/z* = 46) could be observed when employing ^13^CO and H_2_^18^O as the reagents, respectively, due to the WGSR (Figs S27 and S28). In addition, no signals of ^13^CO_2_ (*m/z* = 45) or C^18^O_2_ (*m/z* = 46, 48) could be detected when employing ^13^C_2_H_6_ or ^18^O_2_ as the reagents, further excluding the contribution of O_2_ and ethane towards production of CO_2_ (Figs S29 and S30). In brief, the WGSR process can be well confirmed in the complex reaction system of C_2_H_6_-O_2_-CO-H_2_O for ethane oxidation.

Kinetic measurements were then performed for deep insight into the mechanism of ethane oxidation. As shown in Fig. S31, when employing ^18^O_2_ as the reagent, the kinetic isotope effect (KIE) value of 0.96 was calculated from the overall oxygenates, implying that the activation of O_2_ should not be the rate-determining step. In contrast, when employing H_2_^18^O as the reagent, the KIE values of 1.36 and 1.46 were calculated from the overall oxygenates and acetate acid, respectively, suggesting the possible involvement of H_2_O in the rate-determining step (Fig. S32). Collectively, these results provide comprehensive molecular-level insights into the mechanism of ethane oxidation over the homogeneous Pd(II) catalyst, highlighting the critical roles of both the WGSR and *in situ* generation of reactive oxygen species in achieving high ethane conversion and maintaining high acetate acid selectivity.

### Understanding and refining of the ethane oxidation system

**Figure 3.**
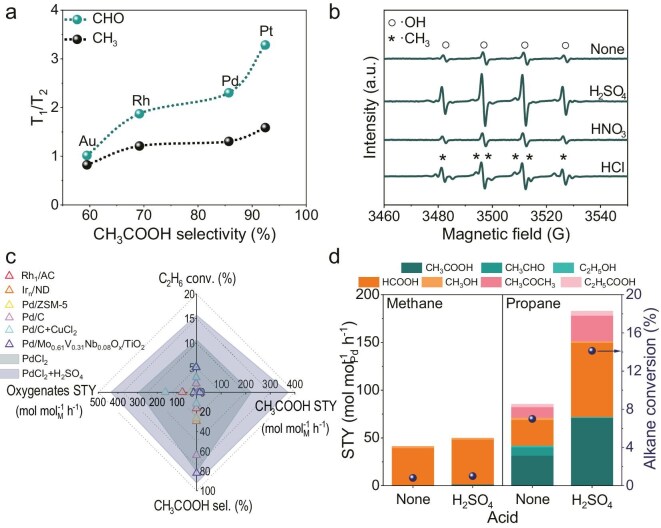
Refining of the ethane oxidation system. (a) Acetate acid selectivity in ethane oxidation versus the T_1_/T_2_ ratio of acetaldehyde measured by NMR. (b) EPR spectra of the reaction solution after ethane oxidation with different acid addition. Reaction conditions: 2.9 μmol Pd, 1.2 MPa O_2_, 1 MPa CO, 2 MPa C_2_H_6_, 10 mL H_2_O, 0 or 0.3 mol L^−1^ H_2_SO_4_/HNO_3_/HCl, 473 K, 2 h. (c) Catalytic performance of PdCl_2_ in comparison with various noble metal catalysts reported for the selective oxidation of ethane. (d) Catalytic performance of PdCl_2_ in the selective oxidation of methane and propane. Reaction conditions: methane, 2.9 μmol Pd, 1.2 MPa O_2_, 1 MPa CO, 2 MPa CH_4_, 10 mL H_2_O, 0 or 0.3 mol L^−1^ H_2_SO_4_, 473 K, 2 h; propane, 2.9 μmol Pd, 0.5 MPa O_2_, 0.4 MPa CO, 0.8 MPa C_3_H_8_, 10 mL H_2_O, 0 or 0.3 mol L^−1^ H_2_SO_4_, 473 K, 2 h.

To understand the difference in acetate acid selectivity across various metal species, the relaxation time including longitudinal relaxation time (T_1_) and transverse relaxation time (T_2_) of ethanol and acetaldehyde were tested by NMR (Figs S33–S36). The T_1_/T_2_ ratio of ethanol demonstrates approximate equivalence while the T_1_/T_2_ ratio of acetaldehyde clearly correlates with the selectivity towards acetate acid. Typically, a higher T_1_/T_2_ ratio indicates stronger adsorption of acetaldehyde on Pd species, which in turn favors its further oxidation to acetate acid (Fig. 3a and Fig. S37). On the other hand, Pd(II) species are highly efficient in catalyzing the WGSR and *in situ* H_2_O_2_ and ·OH formation, and therefore, show high activity in ethane C–H bond cleavage. Considering both these two aspects, PdCl_2_ appears to be the best homogeneous catalyst for the oxidation of ethane to acetate acid in the reaction system of C_2_H_6_-O_2_-CO-H_2_O, showing the highest acetate acid STY (Fig. 1a).

Subsequently, the C_2_H_6_-O_2_-CO-H_2_O reaction system for ethane oxidation was refined by adding various mineral acids [25]. The catalytic performance of PdCl_2_ and the intensity of **·**OH formed were focused on. As shown in Fig. S38, H_2_SO_4_ showed positive impacts on both oxygenated yield and acetate acid selectivity, corresponding to the significantly enhanced **·**OH intensity (Fig. 3b). In contrast, the addition of HCl resulted in minimal oxygenate production, likely due to high concentrations of Cl⁻ coordinating with Pd(II) sites and thus inhibiting the activation of methyl radicals (·CH_3_), as supported by the detection of ·CH_3_ in the EPR spectrum (Fig. 3b). Given the superior performance observed with the addition of H_2_SO_4_, the impact of acid concentration on the time-on-stream catalytic performance was investigated. As shown in Fig. S39, the highest acetic acid STY of 372.7 mol mol_Pd_^−1^ h^−1^ (average value at 2 h) was achieved at the H_2_SO_4_ concentration of 0.3 mol L^−1^, with ethane conversion as high as 15.7%. Surprisingly, acetate acid selectivity gradually rose with increasing H_2_SO_4_ concentration, reaching 94.3% with a H_2_SO_4_ concentration of 0.4 mol L^−1^. This high acetate acid selectivity might relate to the promoted oxidation of acetaldehyde to acetate acid, as indicated by the decline in the selectivity towards acetaldehyde. At the same time, the reaction orders of H^+^ concentrations based on oxygenate and acetic acid formation were calculated to be 0.10 and 0.12, respectively (Fig. S40). The near zero reaction order of H^+^ explains the limited impact of protons on ethane oxidation performance, in accordance with the results shown in Fig. S39. On this basis, the PdCl_2_ demonstrated state-of-the-art performance in ethane oxidation in terms of ethane conversion, oxygenate and acetate acid STY, and acetate acid selectivity (Fig. 3c and Table S4). Given the excellent oxidation performance achieved with the homogeneous PdCl_2_ catalyst, the general applicability of this system was investigated for other light alkanes. As shown in Fig. 3d, the activity in propane oxidation is comparable to that in ethane oxidation (more formic acid byproduct formation due to cleavage of the C–C bond in propane molecules), while the oxidation of methane is significantly limited due to its highly symmetrical and stable structure.

### Reaction mechanism from theoretical calculations

**Figure 4.**
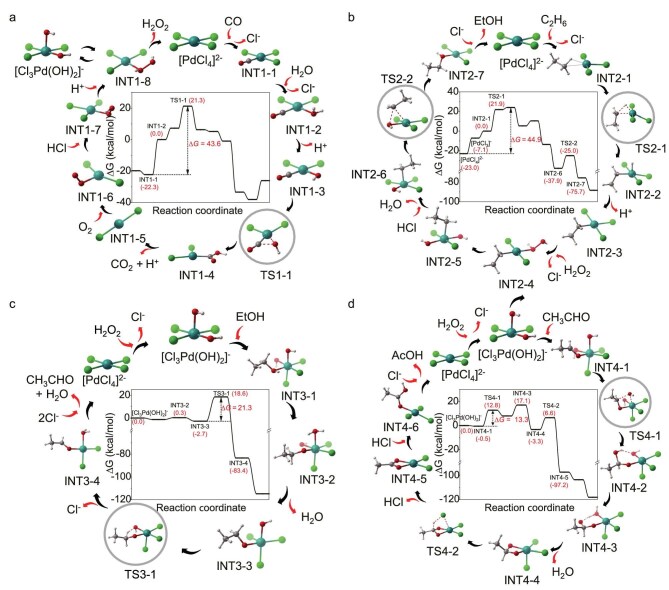
Reaction mechanism of ethane oxidation from DFT calculations. The Gibbs free energy profiles and reaction pathways of H_2_O_2_ generation (a), ethanol formation (b), acetaldehyde formation (c), and acetate acid formation (d). The energy references for each pathway are **INT1-2** for (a), **INT2-1** for (b), and [Cl_3_Pd(OH)_2_]^−^ for both (c) and (d), respectively.

To bridge experimental observations with intrinsic reaction mechanisms, DFT calculations were performed to comprehend the reaction pathway of ethane oxidation to acetic acid as well as to identify the active species. First of all, the coordination situation of Pd species among the reaction solution under various conditions was analyzed by mass spectrometry, as shown in Fig. S41. It is clearly seen that Pd species are coordinated with OH and Cl species. Based on these observations, the DFT-calculated mechanism divides the formation of acetate acid into four sequential processes, involving the generation of H_2_O_2_, ethanol formation, acetaldehyde formation, and eventually acetate acid formation. H_2_O_2_ plays a critical role in this pathway, serving both as an oxygen source and as the terminal oxidant. The Gibbs free energy profiles for the key steps are presented in Fig. 4, Fig. S42, and Table S5. Notably, the calculation results reveal that the H⁺ required for the protonation steps is supplied by HCl present in the PdCl_2_ solution, while Cl⁻ facilitates the subsequent deprotonation steps. These mechanism insights are in good agreement with our experimental observations, bridging the gap between theory and practice in the ethane oxidation process.

The pathway of H_2_O_2_ generation can be divided into the reduction process of Pd(II) by CO and the oxidation process of Pd(0) by O_2_, as shown in Fig. 4a. The reaction initiated with **[PdCl_4_]^2−^** (Fig. S43) undergoing sequential ligand exchanges with CO and H_2_O to form an active substrate **INT1‑2**, followed by deprotonation and C–O bond formation *via*  **TS1‑1** (14.0 kcal/mol). Notably, the process of ligand exchange between CO and Cl^−^ is exoergic, indicating that CO is more easily coordinated on the Pd center. Therefore, elevated CO pressures may lead to greater competition between CO and other species for coordination on the Pd center, which will suppress the reaction. This is consistent with the experimental observations that using CO at high pressures lead to declines in product yield (Fig. 1c). Although the ligand exchange between Cl^−^ and H_2_O is endothermic, requiring a free energy of 22.3 kcal/mol, the aqueous phase environment and high reaction temperature (473 K) both drive the reaction equilibrium of **INT1-1 +** H_2_O → **INT1-2** + Cl^−^ to the right. As evidenced experimentally, Pd species are predominantly coordinated by both Cl and OH rather than just Cl (Fig. S41), where OH should come from H_2_O deprotonation. Next, **INT1‑4** releases CO_2_ and H^+^ to yield Pd(0) species, which react with O_2_ to form Pd–OOH species **INT1‑7** by protonation. After further protonation, the Pd complex can release H_2_O_2_ and return to **[PdCl_4_]^2−^** by ligand exchange, or break the O–O bond to obtain corresponding Pd(IV) species for further ethane oxidation processes. In summary, during the formation of H_2_O_2_, the Pd(II) species are first reduced to Pd(0) species by oxidizing CO, and subsequently re-oxidized back to Pd(II) by O_2_, concomitantly generating H_2_O_2_. The rate-determining step (RDS) of this pathway is C–O bond formation (**TS1‑1**) and the overall energy is 43.6 kcal/mol (from **INT1‑1** to **TS1‑1**), including energy of the ligand exchange of H_2_O with Cl^−^, protonation, and C–O bond formation.

Subsequently, the formation of ethanol involves sequential key steps of coordination of ethane to the Pd center, oxidative addition of ethane on the Pd catalyst, coordination of H_2_O_2_ to the Pd center, decomposition of H_2_O_2_, and reductive elimination to form ethanol (Fig. 4b). One major difference of this process compared with formation of acetaldehyde (Fig. 4c) and acetate acid (Fig. 4d) is that ethane initially interacts with Pd(II) instead of Pd(IV). The RDS of this process is the oxidative addition of ethane to the Pd catalyst with overall energy of 44.9 kcal/mol (from **[PdCl_4_]^2−^** to **TS2‑1**, 21.9 kcal/mol). Note that the Gibbs free energy of **INT2‑2** is higher than that of **TS2‑1**, due to the higher electronic energy, from the single point energy calculation, of **INT2-2** than that of **TS2-1**, while IRC calculation indicates that **TS2‑1** can correctly point towards **INT2-2** (Fig. S44). Similar to the generation of H_2_O_2_, the ligand exchange of ethane with Cl^−^, requiring free energy of 23.0 kcal/mol, makes the greatest contribution. Due to the high temperature and high ethane pressure employed, this exchange process is also feasible.

The formation of acetaldehyde basically consists of the coordination of ethanol deprotonation of the CH_3_CH_2_OH moiety with the assistance of the OH ligand, proton transfer from the CH_3_CH_2_O moiety to the OH ligand to form the CH_3_CHO complex, and ligand exchange with Cl^−^ to release acetaldehyde, as shown in Fig. 4c. The rate-determining step of the acetaldehyde formation pathway is the proton transfer *via*  **TS3-1** and the overall activation energy of 21.3 kcal/mol, which is much lower than H_2_O_2_ generation (Fig. 4a) and ethanol formation (Fig. 4b). It can be seen that when CO, H_2_O, and ethane coordinate to **[PdCl_4_]^2^^−^**, they all need to undergo ligand exchange with the ligand Cl^−^ on Pd, and these ligand exchange steps require high energy absorption. Therefore, from the perspective of chemical equilibrium, lower Cl^−^ concentrations in the system will facilitate these ligand exchange processes. On the other hand, many protonation steps in the reaction require the assistance of H^+^, and many deprotonation steps require the assistance of bases (such as Cl^−^). These protonation and deprotonation steps mostly release energy, meaning that they are more likely to occur. Therefore, the reaction may be promoted as the concentration of Cl^−^ in the reaction system decreases and the concentration of H^+^ increases. This is also consistent with the fact that adding H_2_SO_4_ in addition to equimolar HCl present in PdCl_2_ solution can increase the product yield, while high concentrations of HCl inhibit the reaction (Fig. S38).

Different from previous oxidation processes, the formation of the C–O bond occurs before C–H bond cleavage due to the presence of the C=O bond and the preference for nucleophilic attack during the formation of acetate acid, as shown in Fig. 4d. It is worth noting that ethanol competes with acetaldehyde for coordinating with the Pd center, which finally leads to the competitive formation of acetaldehyde and acetate acid. The nucleophilic attack is the RDS of this process with an energy barrier of only 13.3 kcal/mol, indicating that formation of acetate acid along this pathway can proceed smoothly. The DFT calculation results show that the activation energies for formation of acetaldehyde and acetate acid are lower in comparison with those for H_2_O_2_ generation and ethanol formation. Therefore, the formation rate of acetate acid is primarily determined by the formation rates of H_2_O_2_ and ethanol.

## CONCLUSION

In this study, palladium-catalyzed selective oxidation of ethane to acetate acid is developed as a promising route for ethane conversion and upgrading. It is found for the first time that a simple PdCl_2_ catalyst can efficiently catalyze the selective oxidation of ethane to acetate acid in the reaction system of C_2_H_6_-O_2_-CO-H_2_O. The reaction starts from the WGSR, followed by *in-situ* H_2_O_2_ and **·**OH formation for ethane activation and selective oxidation. The reaction system has been thoroughly investigated and optimized through the combination of kinetic and spectroscopic analyses with isotope labelling, and the advantages of the reaction system are definitely disclosed. In the refined reaction system catalyzed by PdCl_2_ with the assistance of 0.3 mol L^−1^ H_2_SO_4_, high ethane conversion of 15.7% and high acetate acid selectivity of 92.1% can be simultaneously achieved at 473 K after reaction for 2 h, deriving a state-of-the-art acetate acid STY of 372.7 mol_CH3COOH_ mol_Pd_^−1^ h^−1^ which is distinctly higher than previous reports. DFT calculations demonstrate the *in-situ* generation of H_2_O_2_ and the stepwise oxidation of ethane towards acetic acid in the system of C_2_H_6_-O_2_-CO-H_2_O catalyzed by the **[PdCl_4_]^2^^−^** motif, providing theoretical support to the experimental observations. The development of a unique reaction and catalytic system presented herein may unlock the potential of natural gas upgrading under mild conditions.

## MATERIALS AND METHODS

The Pd(II) species catalyst was obtained by dissolving solid PdCl_2_ in a certain amount of water, while adding traces of hydrochloric acid to promote dissolution.

Ethane oxidation reactions were performed in a 50-mL high-pressure stainless-steel autoclave reactor with a magnetic stirrer. In a typical reaction, the above prepared PdCl_2_ catalyst and 10 mL deionized water were added into the autoclave. After purging with ethane three times, the reactor was pressurized with a gas mixture of ethane, oxygen, and carbon monoxide. When there was no further variation in pressure, the reactor was heated to the desired temperature and kept for a certain time (0.5–2 h) at a speed of 1000 r/min. When the reaction finished, the reactor was quickly moved into an ice bath.

The gas products were collected using a gas-sampling bag and analyzed using gas chromatography (GC 7900), equipped with a Porapak Q packed column and a nickel conversion furnace, and further confirmed by the mass spectrometer (Pfeiffer Omnistar GSD 320). The liquid products were analyzed by ^1^H NMR (Bruker AVANCE III 400 spectrometer). Typically, the obtained liquid product solution (500 μL) was mixed with 100 μL dimethyl sulfoxide (DMSO) internal standard (diluted to 900 ppm by D_2_O). The products were quantified by standard curves using the correlation between the ratio of the peak area of oxygenate products to the peak area of DMSO. At the same time, the main product can be detected by gas chromatography-mass spectrometry (Shimadzu GCMS-QP2010 SE) including acetic acid, acetaldehyde, and formic acid.

Ethane oxidation reactions with ^13^C_2_H_6_, ^13^CO, H_2_^18^O, C^18^O, and ^18^O_2_ were also conducted under similar conditions in the autoclave reactor as mentioned above as was the analysis of products.

## Supplementary Material

nwaf355_Supplemental_File
