# Supplementary material for: Palladium-catalyzed selective oxidation of ethane to acetate acid
Source: Natl Sci Rev. 2025 Aug 23;12(11):nwaf355. doi: 10.1093/nsr/nwaf355 (PMC12596251; doi:10.1093/nsr/nwaf355)
Supplement: nwaf355_Supplemental_File [file nwaf355_supplemental_file.pdf]

Supplementary Materials for

Palladium-catalyzed selective oxidation of ethane to acetate acid

Ting Li[1#], Baotong Huang[1#], Weijie Li[1#], Xin Deng[1*], Yuchao Chai[1], Guangjun Wu[1], Xiufang Xu[1*], and Landong Li[1,2*]

[1] Key Laboratory of Advanced Energy Materials Chemistry of Ministry of Education, College of Chemistry, Nankai University, Tianjin 300071, P.R. China

[2] Frontiers Science Center for New Organic Matter, Nankai University, Tianjin 300071, P.R. China

[#]: These authors contributed equally.

Corresponding emails: dengx@nankai.edu.cn; xxfang@nankai.edu.cn; lild@nankai.edu.cn

This file includes:

Materials and Methods

Supplementary Text

Figures S1 to S44

Tables S1 to S5

References 30 to 43

Materials and Methods

Reagents and gases.

Palladium nitrate (Pd≥18.09%, $Pd(NO_3)_2 \cdot 2H_2O$), Palladium chloride (Pd≥59%, $PdCl_2$), Palladium sulfate (Pd≥44%, $PdSO_4$), Palladium acetate (98%, $Pd(CH_3COO)_2$), Palladium hydroxide (20%, $Pd(OH)_2$), Chloroauric acid (Au≥48%, $HAuCl_4$), Chloroplatinic (Pt≥37.5%, $H_2PtCl_6 \cdot 6H_2O$), Ruthenium chloride (98%, $RuCl_3 \cdot 3H_2O$), Chloroiridic acid (Ir≥39%, $H_2IrCl_6 \cdot xH_2O$), Nickel nitrate (98%, $Ni(NO_3)_2 \cdot 6H_2O$), Cobalt nitrate ($Co(NO_3)_2 \cdot 6H_2O$), Copper nitrate (99.9%, $Cu(NO_3)_2 \cdot xH_2O$), Rhodium Chloride (Rh≥38.5%, $RhCl_3 \cdot 3H_2O$), Ferric nitrate (≥98%, $Fe(NO_3)_3 \cdot 9H_2O$), Indium nitrate (99.9%, $In(NO_3)_3 \cdot 4H_2O$).

Sulfuric acid (95-98%, H_2SO_4), Hydrochloric acid (36-38%, HCl), Nitric acid (65-68%, HNO_3), Acetic acid (≥99.7%, CH_3COOH), Ethanol (99.7%, CH_3CH_2OH), Acetaldehyde (99.5%, CH_3CHO), Methanol (99.9%, CH_3OH), Formic acid (96%, HCOOH), Hydrogen peroxide (30%, H_2O_2), Dimethyl sulfoxide (≥99.8%, C_2H_6OS), 5,5-dimethyl-1-pyrroline N-oxide (≥98%, $C_6H_{11}NO$) were supplied by Aladdin. Oxygen (100%, O_2), ethane (100%, C_2H_6), carbon monoxide (100%, CO) were supplied by Dalian Special Gas Co. Ltd. The $^{13}C_2H_6$ (^{13}C, 99%), ^{13}CO (^{13}C, 99%), $C^{18}O$ (^{18}O, 99%) and $^{18}O_2$ (^{18}O, 99%) were purchased from Guangzhou Yuejia Gas Co. Ltd. D_2O (^2H, 99.99%) and $H_2{}^{18}O$ (^{18}O, 99.99%) was purchased from Guangzhou Yuejia Gas Co. Ltd. All materials and reagents were used directedly without purification.

Catalytic testing of ethane oxidation

Ethane oxidation reactions were performed in a 50 mL high-pressure stainless-steel autoclave reactor with a magnetic stirrer. In a typical reaction, $PdCl_2$ dissolved in equimolar HCl (2.9 μmol Pd) and 10 mL deionized water were added into the autoclave. After purging with ethane three times, the reactor was pressured with a gas mixture of ethane, oxygen and carbon monoxide. After no variation in pressure, the reactor was heated to desired temperature and keep a certain time at a speed of 1000 rpm. When the reaction finished, the reactor was moved into an ice bath rapidly. The gas products were collected using a gas-sampling bag and analyzed using gas chromatography (GC 7900), equipped with a Porapak Q packed column, a nickel conversion furnace and further confirmed by the mass spectrometer (Pfeiffer Omnistar GSD 320). The liquid products were analyzed by ^1H NMR (Bruker

AVANCE III 400 spectrometer). Typically, the obtained liquid product solution (500 μL) was mixed with 100 μL dimethyl sulfoxide internal standard (DMSO, diluted to 900 ppm by D_2O). The products were quantified by standard curves using the correlation between the ration of the peak area of oxygenates products to the peak area of DMSO (**Figures S1-S6**). The chemical shifts and splitting for the protons of different products are shown in **Table S3**. A standard ^{1}H NMR spectrum is shown in **Figure S6**. At the same time, the main product can be detected on Gas chromatography-mass spectrometry (GC-MS) including acetic acid, acetaldehyde, formic acid (**Figure S15**).

Ethane oxidation using hydrogen peroxide (H_2O_2) as oxidant was performed using the same procedure. $PdCl_2$ dissolved in small amount of HCl (2.9 μmol Pd) and 10 mL H_2O_2 (0.02 mol L^{-1}) were added into the autoclave. After purging with ethane three times, the gas mixture of ethane and nitrogen (C_2H_6, 20 bar; N_2, 22 bar was pressured in the autoclave. After no variation in pressure, the reactor was heated to desired temperature and keep it for a desired time. The subsequent steps were the same as the previous ones.

The quantification of H_2O_2 was performed by titrating the solution with acidified $Ce(SO_4)_2$ solution with a concentration of 0.0025 mol/L using Ferroin as an indicator.

Catalytic testing of methane oxidation

Methane oxidation reactions were performed using the same procedure. $PdCl_2$ dissolved in equimolar HCl (2.9 μmol Pd) and 10 mL deionized water were added into the autoclave. After purging with methane three times, the gas mixture of methane, oxygen and carbon monoxide (CH_4, 20 bar; O_2, 12 bar; CO, 10 bar) was pressured in the autoclave. After no variation in pressure, the reactor was heated to desired temperature at a speed of 1000 rpm and keep it for a desired time. The chemical shifts and splitting for the protons of different products are shown in **Table S3**.

Catalytic testing of propane oxidation

Propane oxidation reactions were performed using the same procedure. The gas mixture of propane, oxygen and carbon monoxide (C_3H_8, 8 bar; O_2, 5 bar; CO, 4 bar) was pressured in the autoclave. The chemical shifts and splitting for the protons of different products are shown in **Table S3**.

Electron paramagnetic resonance (EPR)

5,5-dimethyl-1-pyrroline N-oxide (DMPO) spin-trapping electron paramagnetic resonance (EPR) experiments were conducted on a Bruker Emxplus spectrometer (E580-10/12) at room temperature. The parameters for EPR measurements were as follows: scanning width of 100 G, microwave frequency of 9.85 GHz, microwave power of 15 mW microwave frequency of 100 kHz, sweep time of 10.2 s, attenuator of 10 dB. In a typical experiment, 1 mL DMPO-H_2O (0.276 mol/L) were added into 3 mL reaction mixture. The mixed solution was immediately transferred to a capillary tube (diameter: 1 mm, filling liquid height: 5 cm), which was then fixed in the resonant cavity of the spectrometer.

Two-dimensional (2D) ^{1}H-^{13}C heteronuclear singular quantum correlation (HSQC)

For the 2D HSQC NMR, the liquid product solution obtained from the reaction of $^{13}C_2H_6$ (500 μL) was mixed with 100 mL dimethyl sulfoxide internal standard (DMSO, diluted to 900 ppm by D_2O). The 2D HSQC NMR spectra were recorded in HSQC experiments on a Bruker AVANCE III 400 spectrometer. The spectral widths were 4800 and 22000 Hz for the ^{1}H and ^{13}C dimensions, respectively. The number of collected complex points was 2048 for the ^{1}H dimension with a recycle delay of 2 s. The number of transients was 8, 256 times increments were always recorded in the ^{13}C dimension.

^{13}C labeled isotopic experiments

Ethane oxidation reactions with $^{13}C_2H_6$ or ^{13}CO were also conducted under the similar conditions in the autoclave reactor mentioned above. The signals of $^{13}CO_2$, $^{13}CH_3COOH$, $CH_3{}^{13}COOH$, $^{13}CH_3CHO$, $CH_3{}^{13}CHO$, $^{13}CH_3CHO$ (hydrated), $CH_3{}^{13}CHO$ (hydrated), $^{13}CH_3CH_2OH$, $CH_3{}^{13}CH_2OH$, $H^{13}COOH$ and $^{13}CH_3OH$ are located in 124.7, 20.6, 176.8, 30.2, 206.9, 23.3, 88.4, 16.9, 57.6, 166.0 and 49.1 ppm in ^{13}C-NMR spectra, respectively. The liquid products labelled with carbon-13 including $^{13}CH_3{}^{13}COOH$ (*m/z*=62, 46, 44, 16), $^{13}CH_3{}^{13}CHO$ (*m/z*=46, 30, 16) and $H^{13}COOH$ (*m/z*=47, 30) were analyzed through GC-MS. The gas labelled with ^{13}C were analyzed through MS including CO_2 (*m/z*=44) and $^{13}CO_2$ (*m/z*=45).

^{18}O labeled isotopic experiments

Ethane oxidation reactions with $H_2{}^{18}O$, $C^{18}O$ and $^{18}O_2$ were also conducted under the similar conditions in the autoclave reactor mentioned above. The liquid produces labelled with oxygen-18 including $CH_3C^{18}O^{18}OH$ (*m/z*=64, 49, 45), $CH_3CO^{18}OH$ (*m/z*=62, 47), $CH_3C^{18}OOH$ (*m/z*=62, 47), $CH_3CH^{18}O$

(m/z=46, 45, 31), $HC^{18}O^{18}OH$ (m/z=50, 49, 31, 30), $HCO^{18}OH$ (m/z=48, 47) and $HC^{18}OOH$ (m/z=48, 47) were analyzed through GC-MS. The gas labelled with ^{18}O were analyzed through MS including CO_2 (m/z=44), $CO^{18}O$ (m/z=46) and $C^{18}O_2$ (m/z=48).

^{18}O-^{16}O exchange experiments

The reaction of ^{18}O-^{16}O exchange was tested in the autoclave mentioned above. In a typical reaction, $PdCl_2$ dissolved in small amount of HCl (2.9 μmol Pd), 1.5 mmol $CH_3COOH/CH_3CH_2OH/CH_3CHO$, 2 mL $H_2{}^{18}O$ were added into the autoclave. The reactor was pressured with 4.2 MPa N_2. Then the liquid products were analyzed using GC-MS after reaction.

D labeled isotopic experiments

Ethane oxidation reactions with D_2O under the similar conditions in the autoclave reactor mentioned above. The liquid produces labelled with D including CD_3COOD (m/z=64, 46, 18), CD_3CDO (m/z=48, 46, 30, 18) and DCOOD (m/z=48, 46, 30).

H-D exchange experiments

The reaction of H-D exchange was tested in the autoclave mentioned above. In a typical reaction, $PdCl_2$ dissolved in small amount of HCl (2.9 μmol Pd), 1.5 mmol $CH_3COOH/CH_3CH_2OH/CH_3CHO$, 2 mL D_2O were added into the autoclave. The reactor was pressured with 4.2 MPa N_2. Then the liquid products were analyzed using GC-MS after reaction.

Kinetic isotope effects (KIE)

The kinetic isotope effect (KIE) was tested in the autoclave mentioned above.

In the reaction of kinetic isotope effects of $O_2/^{18}O_2$, $PdCl_2$ dissolved in small amount of HCl (2.9 μmol Pd) and 10 mL deionized water were added into the autoclave. After purging with ethane three times, the gas mixture of ethane, oxygen and carbon monoxide (C_2H_6, 5 bar; $O_2/^{18}O_2$, 3 bar; CO, 2.5 bar; N_2 was replenished to 42 bar as a balance gas) was pressured in the autoclave.

In the reaction of kinetic isotope effects of $H_2O/H_2{}^{18}O$, $PdCl_2$ dissolved in small amount of HCl (1.45 μmol Pd) and 1 mL deionized water or water labeled with oxygen-18 were added into the autoclave. After purging with ethane three times, the gas mixture of ethane, oxygen and carbon monoxide (C_2H_6,

20 bar; O_2, 12 bar; CO, 10 bar) was pressured in the autoclave. The liquid products were analyzed by ^1H NMR.

Computational details

All density functional theory (DFT) calculations were carried out with Gaussian16 program [30]. Geometry optimization and frequency analysis were performed using TPSS exchange-correlation functional [31] and 3-zeta basis set ma-def2-TZVP [32-33] in water with the integral equation formalism-polarizable continuum model (IEF-PCM) [34] at 473.15 K and 1 atm. Single point energy calculation were performed using MN15 functional [35] with 3-zeta basis set ma-def2-TZVPP in water with the solvation model based on density (SMD) [36]. Calculations above were carried out under Grimme's empirical dispersion with Becke-Johnson damping [37]. The Gibbs free energy of each species is obtained by adding electronic energy from single point energy calculation and Gibbs free energy correction from frequency analysis. The electronic energy, thermal energy, enthalpy, and free energy calculated at 473.15 K for each steady state point were shown in **Table S5**. For transition states (TSs), the intrinsic reaction coordinate (IRC) calculations [38] were performed and make sure that the TSs are correctly corresponding to the intermediates on both sides. Not only singlet state but also triplet state calculations were performed for some species, and the results indicate that stable state for the majority of species is the singlet state. Therefore, the reaction should process on singlet state.

Figures and tables

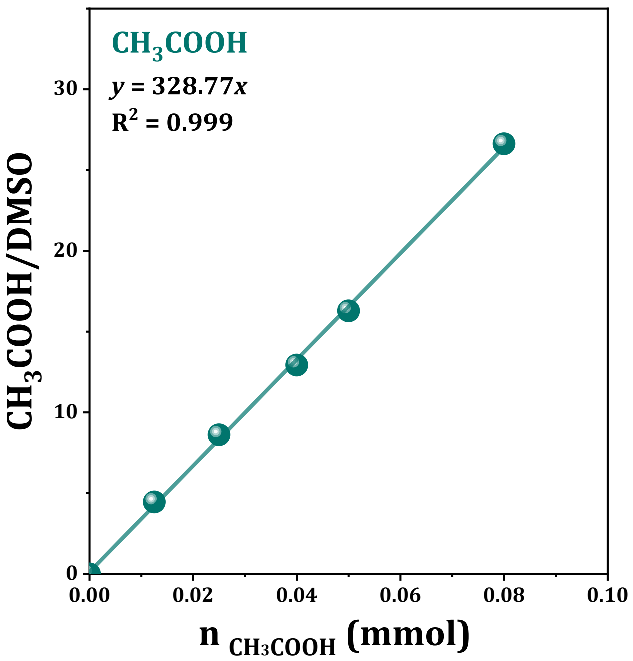

Figure S1. Calibration plots for ^{1}H NMR analysis of acetic acid.

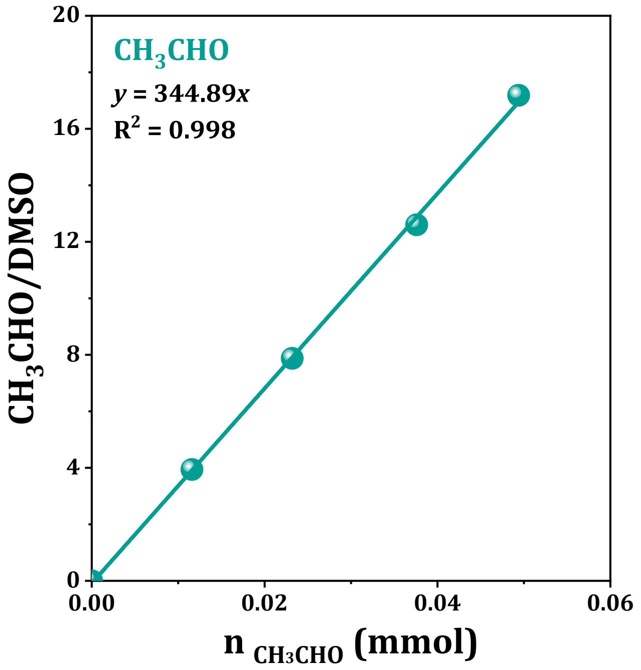

Figure S2. Calibration plots for ^{1}H NMR analysis of acetaldehyde.

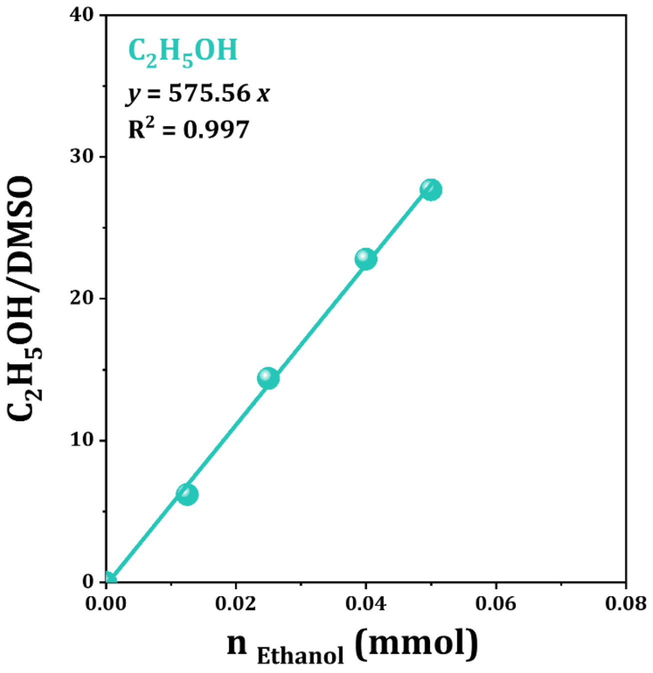

Figure S4. Calibration plots for ^{1}H NMR analysis of ethanol.

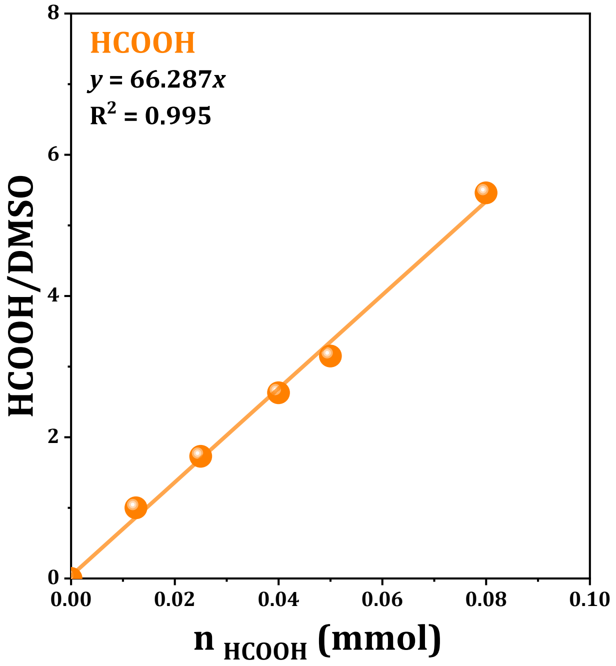

Figure S4. Calibration plots for ^{1}H NMR analysis of formic acid.

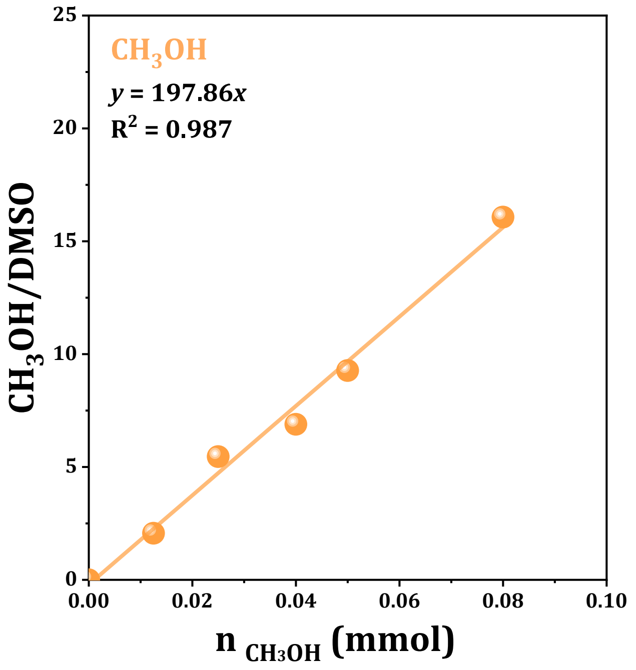

Figure S5. Calibration plots for ^{1}H NMR analysis of methanol.

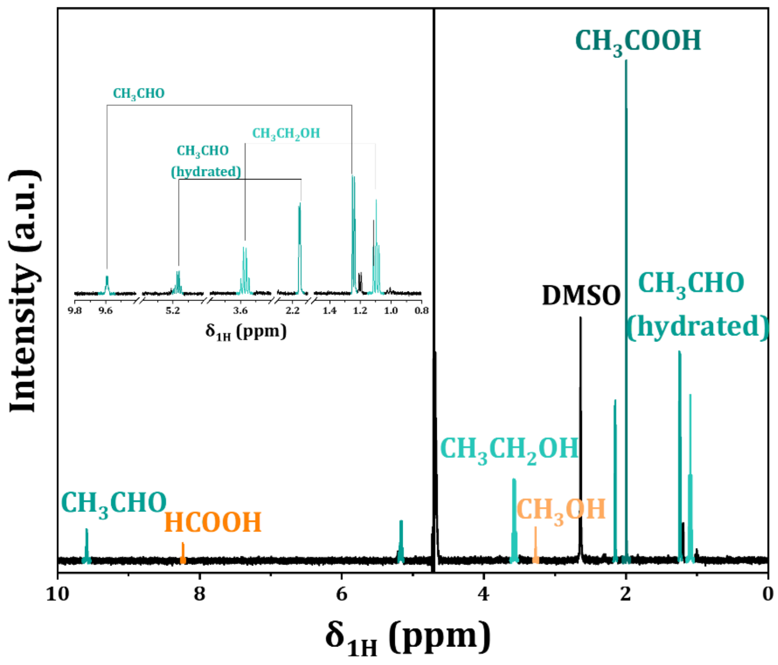

Figure S6. Standard ^{1}H-NMR spectrum of ethane oxidation including acetic acid, acetaldehyde, acetaldehyde (hydrated), ethanol, formic acid, methanol and DMSO.

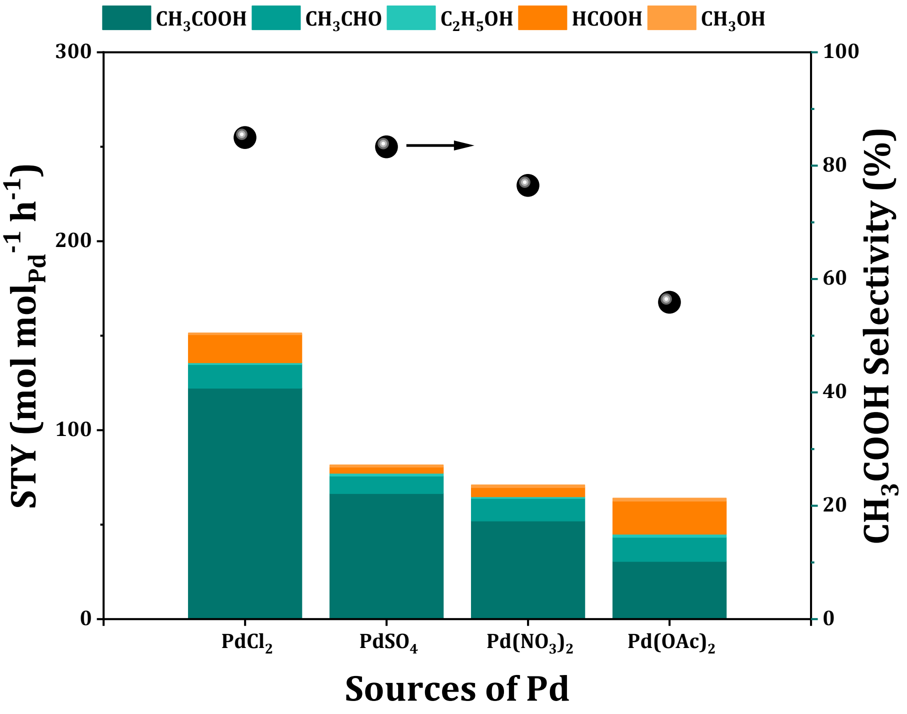

Figure S7. Catalytic performance for ethane oxidation over different Pd-based homogeneous catalysts. Reaction conditions: 2.9 μmol Pd, 0.5 MPa O_2, 0.5 MPa CO, 2 MPa C_2H_6, 10 mL H_2O, 473 K, 2 h.

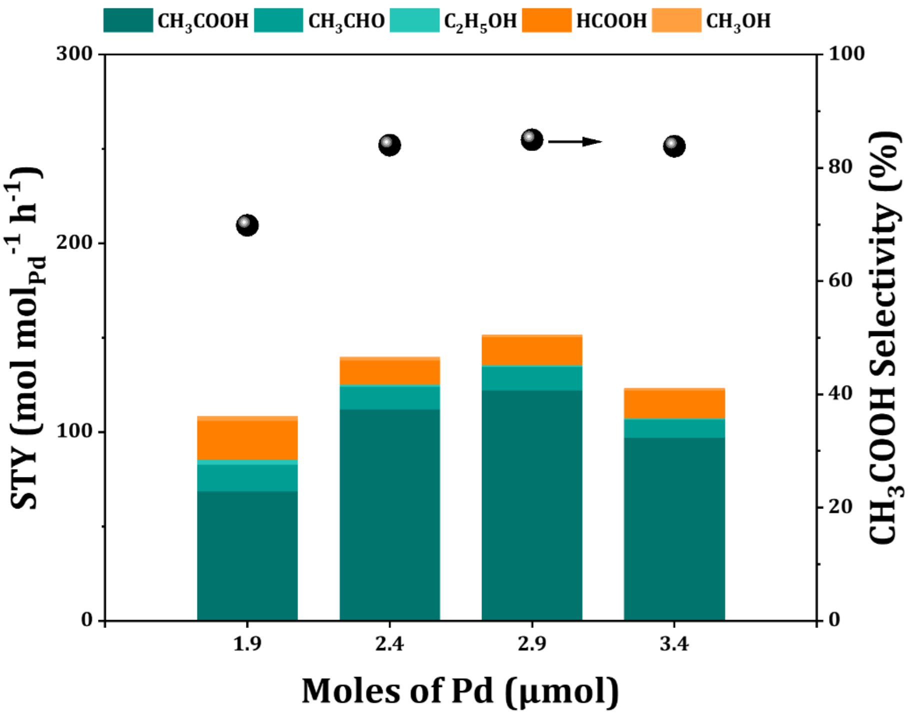

Figure S8. Catalytic performance for ethane oxidation with different Pd dosage. Reaction conditions: 1.9-3.4 μmol Pd, 0.5 MPa O_2, 0.5 MPa CO, 2 MPa C_2H_6, 10 mL H_2O, 473 K, 2 h.

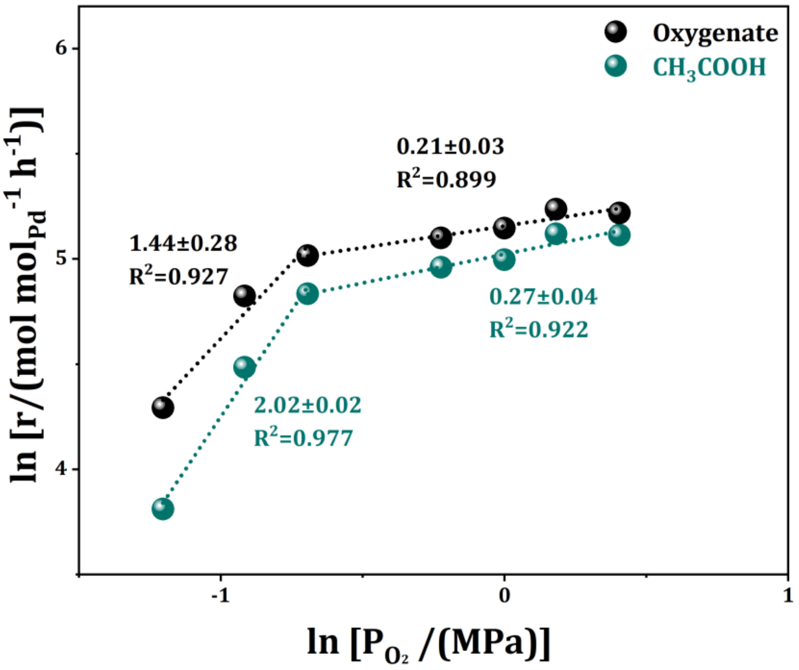

Figure S9. Relationship between O_2 partial pressure and the yield of liquid oxygenates as well as CH_3COOH. Reaction conditions: 2.9 μmol Pd, 0-1.5 MPa O_2, 0.5 MPa CO, 2 MPa C_2H_6, 10 mL H_2O, 473 K, 2 h.

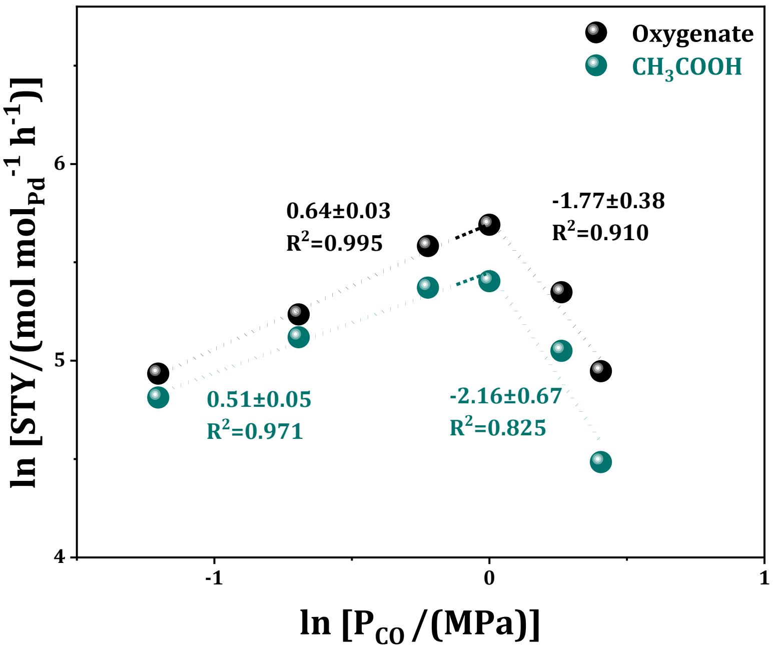

Figure S10. Relationship between CO partial pressure and the yield of liquid oxygenates as well as CH_3COOH. Reaction conditions: 2.9 μmol Pd, 1.2 MPa O_2, 0-1.5 MPa CO, 2 MPa C_2H_6, 10 mL H_2O, 473 K, 2 h.

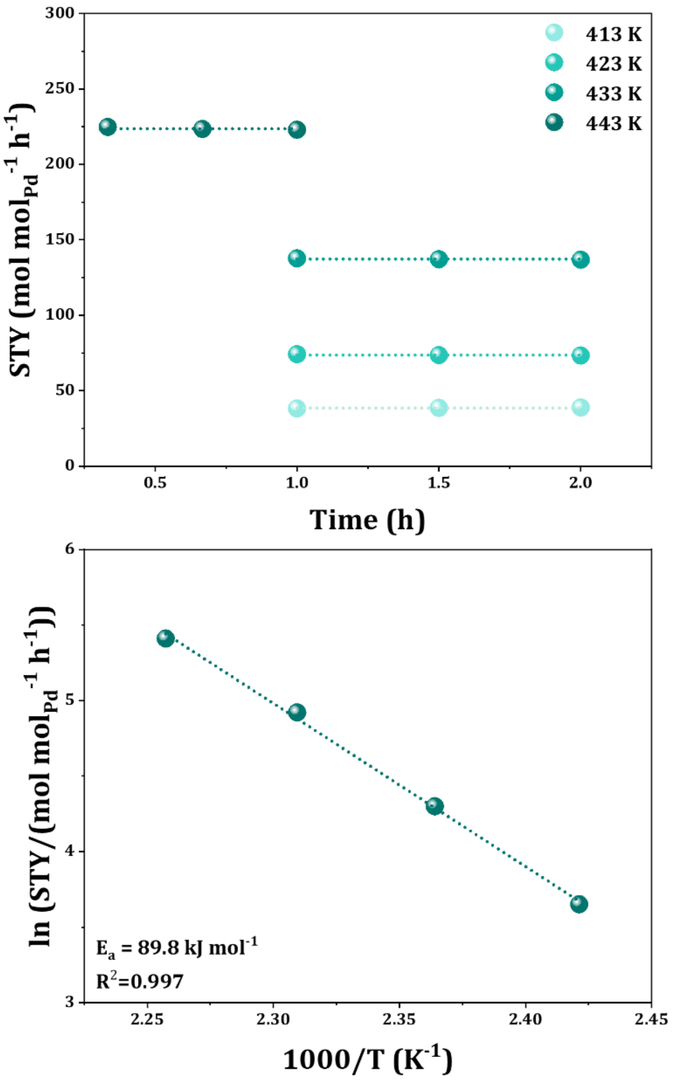

Figure S11. Arrhenius plot for ethane oxidation over $PdCl_2$ catalyst. Reaction conditions: 2.9 μmol Pd, 1.2 MPa O_2, 1 MPa CO, 2 MPa C_2H_6, 10 mL H_2O, 413-443 K, 0.5-2.0 h..

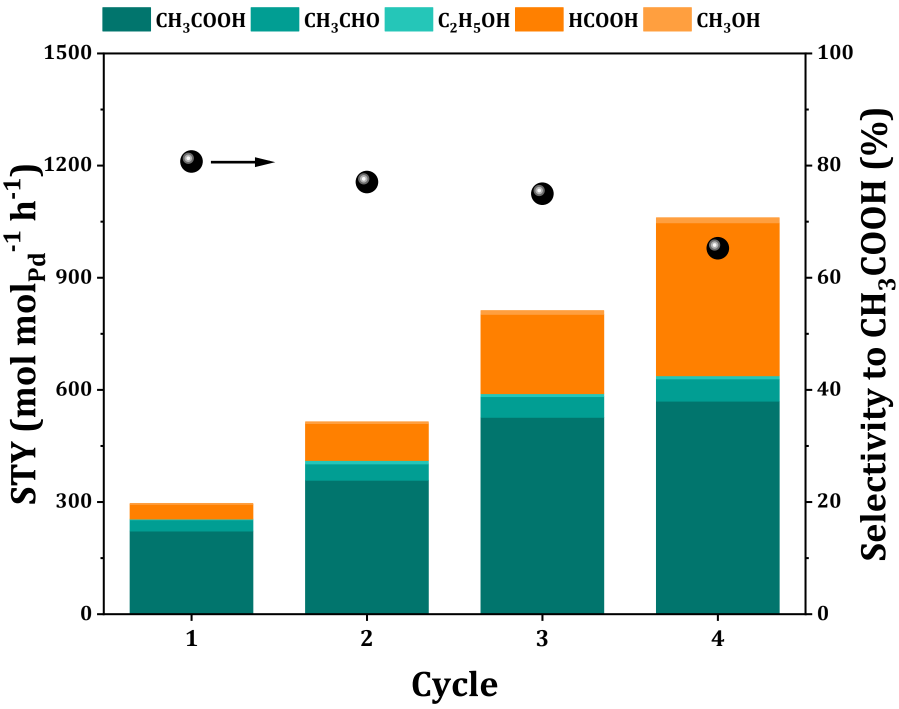

Figure S12. Catalytic performance for successive ethane oxidation over $PdCl_2$ catalyst. Reaction condition: 2.9 μmol Pd, 1.2 MPa O_2, 1 MPa CO, 2 MPa C_2H_6, 10 mL H_2O, 473 K, 2 h. After each reaction, 0.5 mL of the liquid was analyzed using 1H-NMR, then the autoclave was pressed with 1.2 MPa O_2, 1 MPa CO, 2 MPa C_2H_6, for the next cycle.

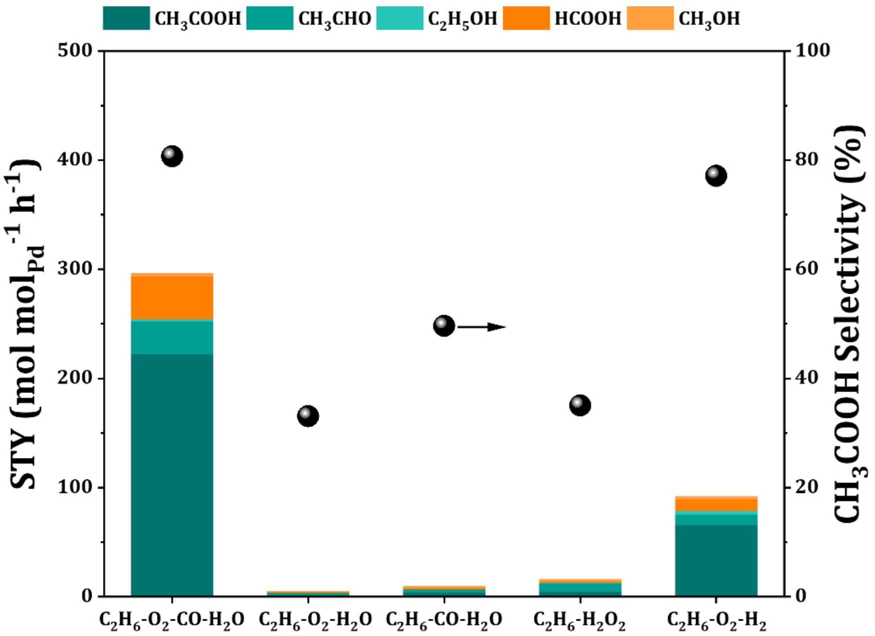

Figure S13. Ethane oxidation over $PdCl_2$ catalyst under various reaction conditions. Reaction conditions: 2.9 μmol Pd, 2.0 MPa C_2H_6, 0 or 1.2 MPa O_2, 0 or 1.0 MPa CO (H_2), 10 mL H_2O or 10 mL H_2O_2 (0.02 mol L^{-1}), 473 K, 2 h. The total pressure was controlled at 4.2 MPa by charging inert gas N_2 if needed.

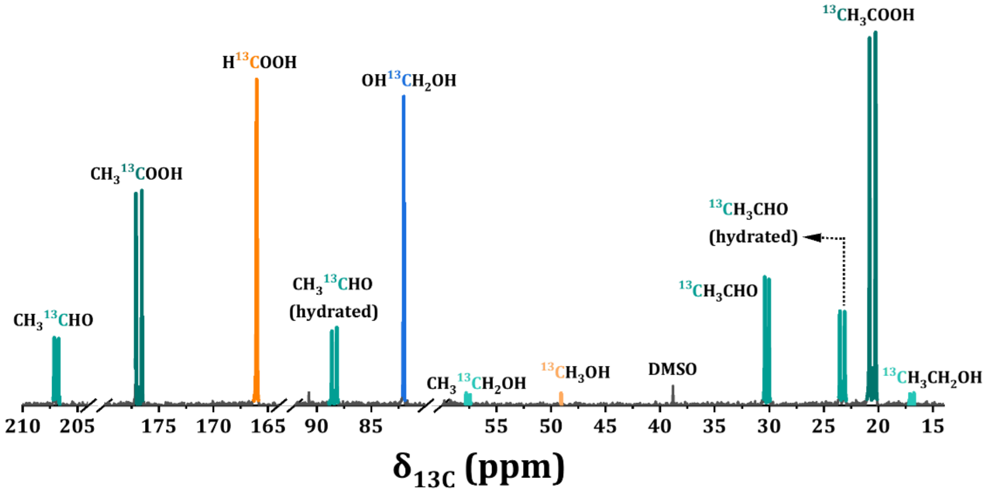

Figure S14. ^{13}C NMR spectrum of oxygenate product in ethane oxidation employing $^{13}C_2H_6$ as the reagent. Reaction conditions: 2.9 μmol Pd, 0.3 MPa O_2, 0.25 MPa CO, 0.4 MPa C_2H_6, 0.1 MPa $^{13}C_2H_6$, N_2 was replenished to 4.2 MPa as a balance gas, 10 mL H_2O, 473 K, 2 h.

Note: There were signal splitting due to the coupling between adjacent ^{13}C nuclei. The signals of acetic acid (δ = 20.6 and 176.8 ppm), acetaldehyde (δ =30.2 and 206.9 ppm), ethanol (δ = 16.9 and 57.6 ppm) and acetaldehyde (hydrated) (δ = 23.3 and 88.4 ppm) appeared as doublets.

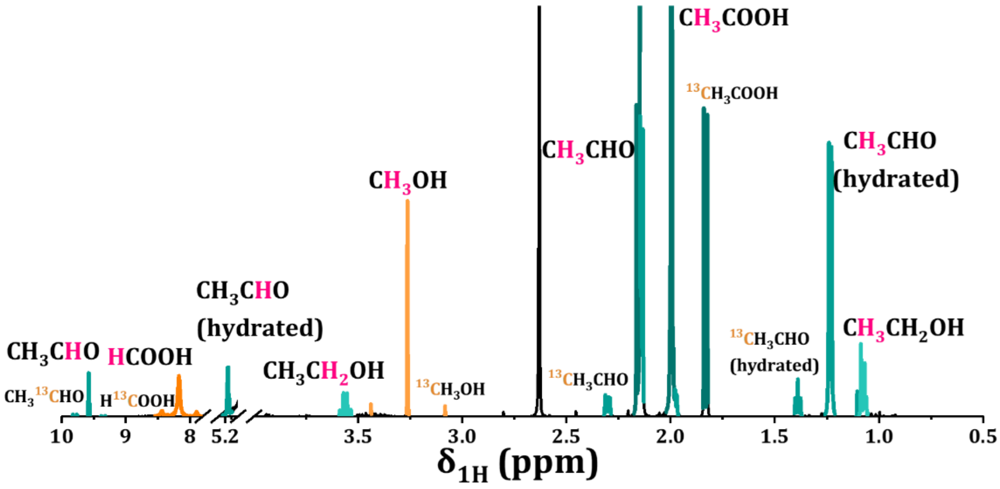

Figure S15. ^{1}H NMR spectrum of isotopic labeling experiments with $^{13}C_2H_6$. Reaction conditions: 2.9 μmol Pd, 0.3 MPa O_2, 0.25 MPa CO, 0.4 MPa C_2H_6, 0.1 MPa $^{13}C_2H_6$, N_2 was replenished to 4.2 MPa as a balance gas, 10 mL H_2O, 473 K, 2 h.

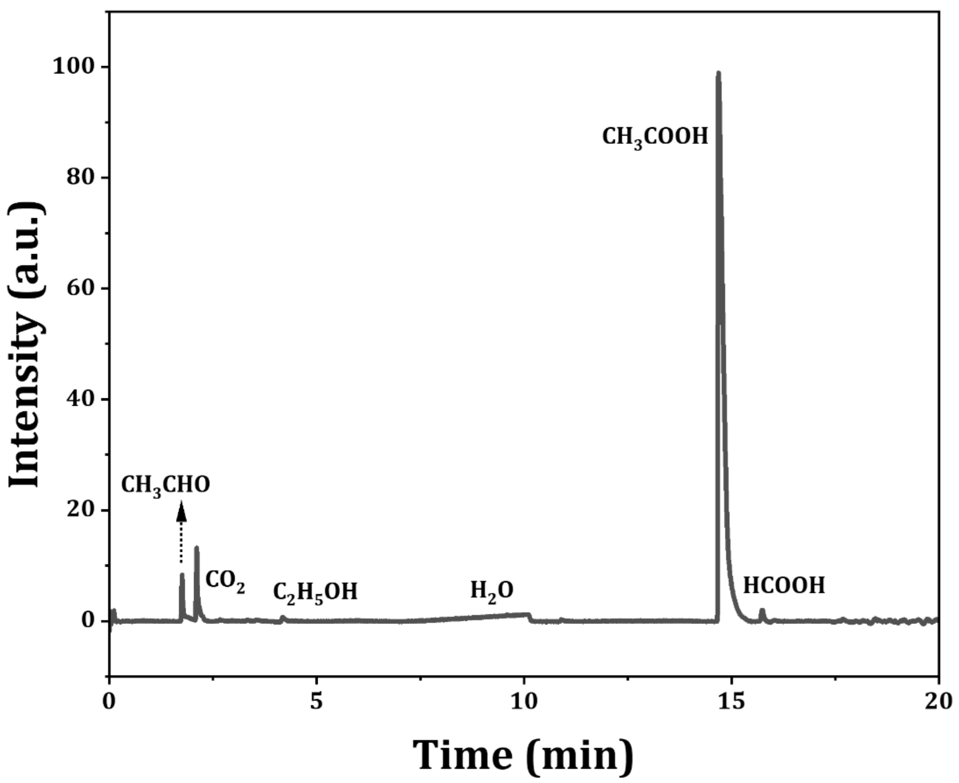

Figure S16. Typical gas chromatography-mass spectrum for the off-line analysis of CH_3COOH, CH_3CHO, C_2H_5OH and HCOOH products from ethane selective oxidation.

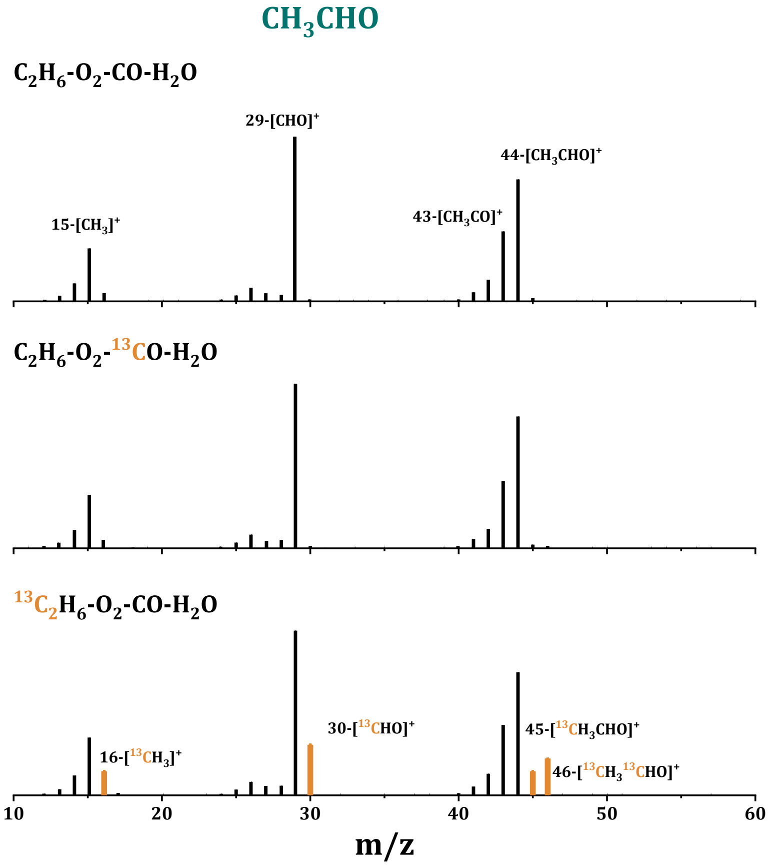

Figure S17. GC-MS analyses of acetaldehyde during ^{13}C isotopic labeling experiments. Reaction conditions: (i) 2.9 μmol Pd, 0.3 MPa O_2, 0.25 MPa ^{13}CO, 0.5 MPa C_2H_6, N_2 was replenished to 4.2 MPa as a balance gas, 10 mL H_2O, 473 K, 2 h; (ii) 2.9 μmol Pd, 0.3 MPa O_2, 0.25 MPa CO, 0.4 MPa C_2H_6, 0.1 MPa $^{13}C_2H_6$, N_2 was replenished to 4.2 MPa as a balance gas, 10 mL H_2O, 473 K, 2 h.

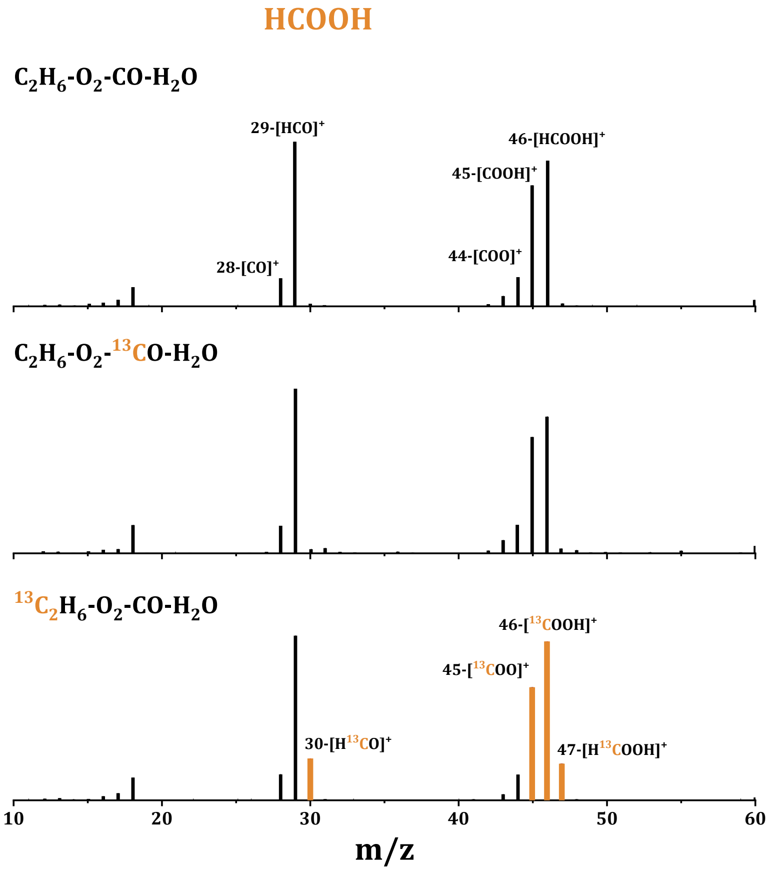

Figure S18. GC-MS analyses of formic acid during ^{13}C isotopic labeling experiments. Reaction conditions: (i) 2.9 μmol Pd, 0.3 MPa O_2, 0.25 MPa ^{13}CO, 0.5 MPa C_2H_6, N_2 was replenished to 4.2 MPa as a balance gas, 10 mL H_2O, 473 K, 2 h; (ii) 2.9 μmol Pd, 0.3 MPa O_2, 0.25 MPa CO, 0.4 MPa C_2H_6, 0.1 MPa $^{13}C_2H_6$, N_2 was replenished to 4.2 MPa as a balance gas, 10 mL H_2O, 473 K, 2 h.

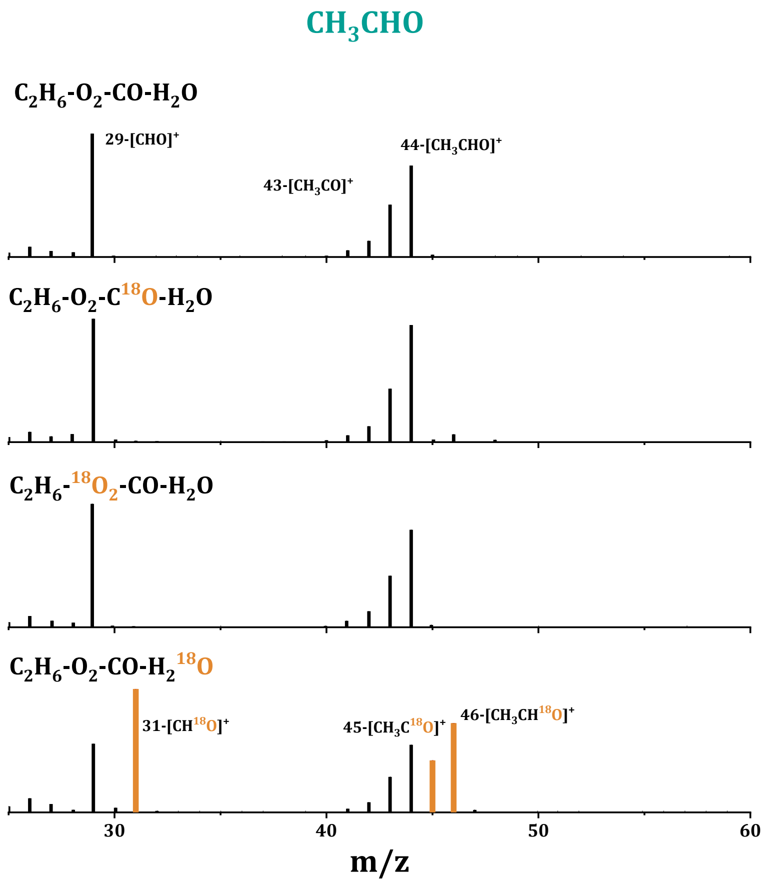

Figure S19. GC-MS analyses of acetaldehyde during ^{18}O isotopic labeling experiments. Reaction conditions: (i) 2.9 μmol Pd, 0.3 MPa O_2, 0.25 MPa $C^{18}O$, 0.5 MPa C_2H_6, N_2 was replenished to 4.2 MPa as a balance gas, 10 mL H_2O, 473 K, 2 h. (ii) 2.9 μmol Pd, 0.3 MPa $^{18}O_2$, 0.25 MPa CO, 0.5 MPa C_2H_6, N_2 was replenished to 4.2 MPa as a balance gas, 10 mL H_2O, 473 K, 2 h; (iii) 2.9 μmol Pd, 1.2 MPa O_2, 1 MPa CO, 2 MPa C_2H_6, 2 mL $H_2{}^{18}O$, 473 K, 2 h.

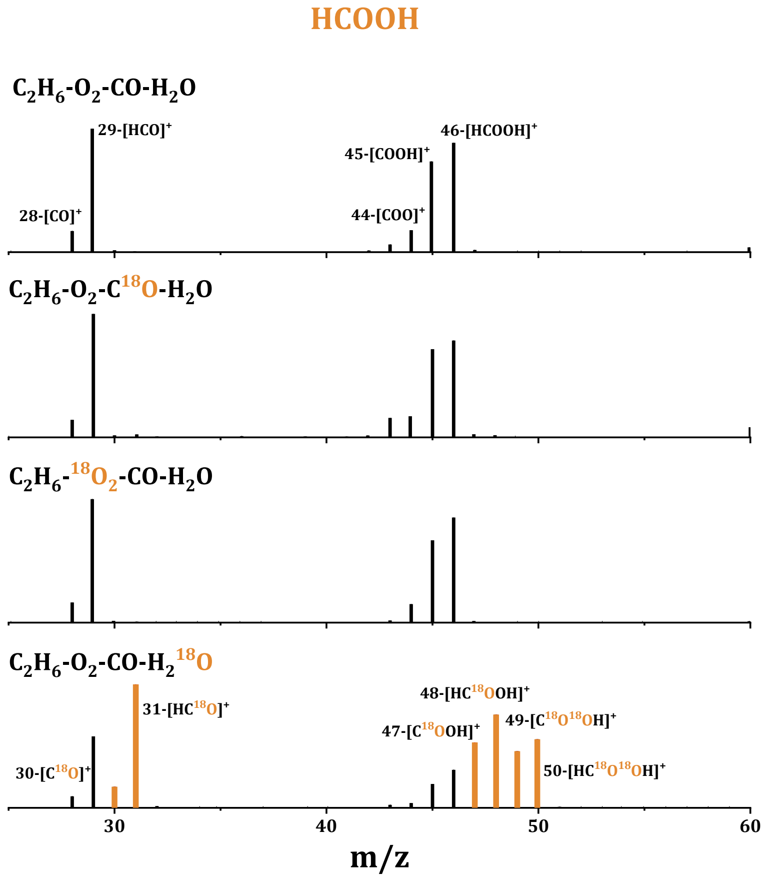

Figure S20. GC-MS analyses of formic acid during ^{18}O isotopic labeling experiments. Reaction conditions: (i) 2.9 µmol Pd, 0.3 MPa O_2, 0.25 MPa $C^{18}O$, 0.5 MPa C_2H_6, N_2 was replenished to 4.2 MPa as a balance gas, 10 mL H_2O, 473 K, 2 h. (ii) 2.9 µmol Pd, 0.3 MPa $^{18}O_2$, 0.25 MPa CO, 0.5 MPa C_2H_6, N_2 was replenished to 4.2 MPa as a balance gas, 10 mL H_2O, 473 K, 2 h; (iii) 2.9 µmol Pd, 1.2 MPa O_2, 1 MPa CO, 2 MPa C_2H_6, 2 mL $H_2{}^{18}O$, 473 K, 2 h.

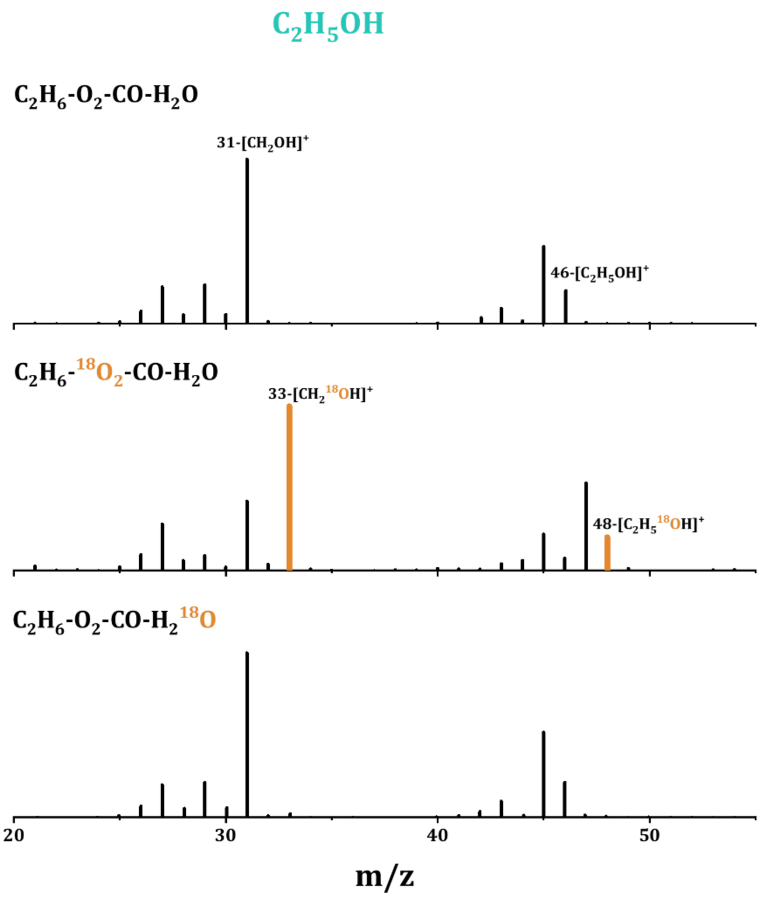

Figure S21. GC-MS analyses of ethanol during ^{18}O isotopic labeling experiments. Reaction conditions: (i) 2.9 μmol Pd, 0.3 MPa $^{18}O_2$, 0.25 MPa CO, 0.5 MPa C_2H_6, N_2 was replenished to 4.2 MPa as a balance gas, 10 mL H_2O, 473 K, 2 h; (ii) 2.9 μmol Pd, 1.2 MPa O_2, 1 MPa CO, 2 MPa C_2H_6, 2 mL $H_2{}^{18}O$, 473 K, 2 h.

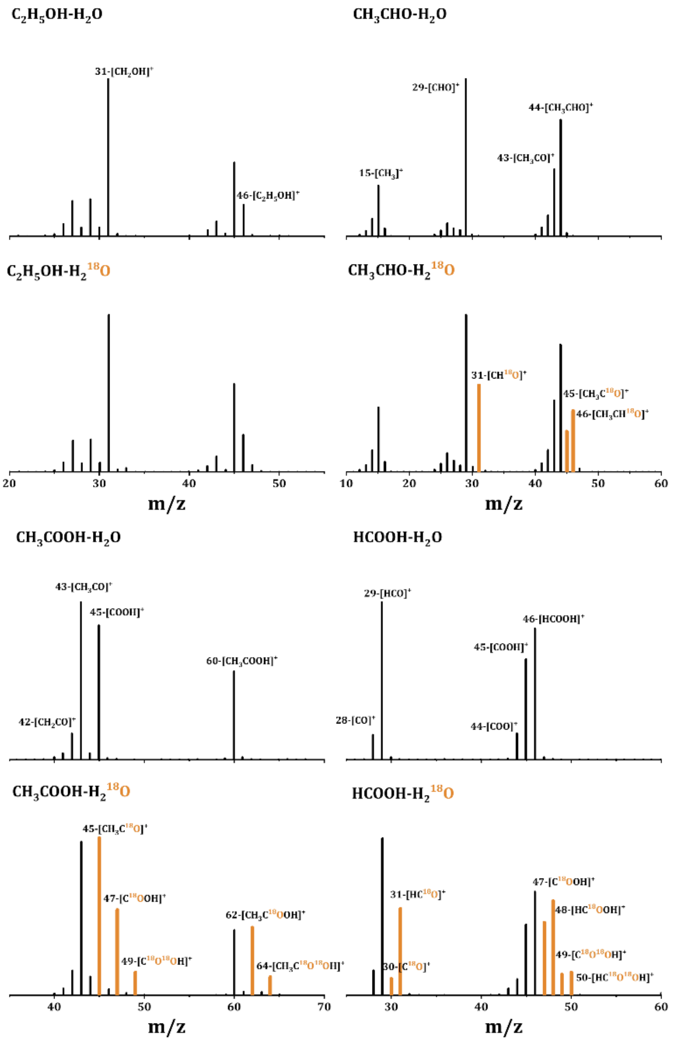

Figure S22. GC-MS analyses of C_2H_5OH, CH_3CHO, CH_3COOH and HCOOH under ^{16}O-^{18}O exchange experiment. Reaction conditions: 2.9 μmol Pd, 1.5 mmol C_2H_5OH/CH_3CHO/ CH_3COOH/HCOOH, 2 mL $H_2{}^{18}O$, 4.2 MPa N_2, 473 K, 2 h.

Note: To further verify whether the ^{16}O-^{18}O exchange occurs in liquid phase under the reaction condition, C_2H_5OH, CH_3CHO, CH_3COOH and HCOOH as well as $H_2{}^{18}O$ were mixed at 473 K with the total pressure of 4.2 MPa N_2 over $PdCl_2$ catalyst. Typical ^{18}O-labelled CH_3COOH, CH_3CHO and HCOOH fragments including $CH_3C^{18}O$ (*m/z*=45), $C^{18}OOH$ (*m/z*=47), $C^{18}O^{18}OH$ (*m/z*=49), $CH_3C^{18}OOH$ (*m/z*=62), $CH_3C^{18}O^{18}OH$ (*m/z*=64) and $CH^{18}O$ (*m/z*=31), $CH_3C^{18}O$ (*m/z*=45), $CH_3CH^{18}O$ (*m/z*=46) and $C^{18}O$ (*m/z*=30), $HC^{18}O$ (*m/z*=31), $C^{18}OOH$ (*m/z*=47), $HC^{18}OOH$ (*m/z*=48), $C^{18}O^{18}OH$ (*m/z*=49), $HC^{18}O^{18}OH$ (*m/z*=48) confirm the ^{16}O-^{18}O exchange between CH_3COOH/CH_3CHO/HCOOH and $H_2{}^{18}O$ which is not observed in the case of C_2H_5OH.

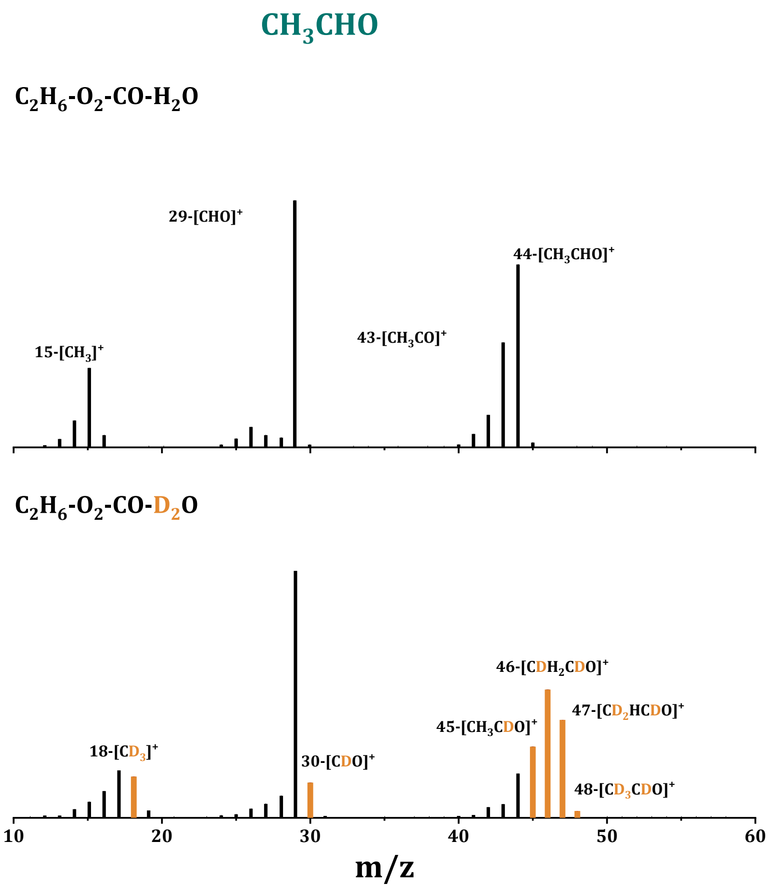

Figure S23. GC-MS analyses of acetaldehyde during D isotopic labeling experiments. Reaction conditions: 2.9 μmol Pd, 1.2 MPa O_2, 1 MPa CO, 2 MPa C_2H_6, 2 mL D_2O, 473 K, 2 h.

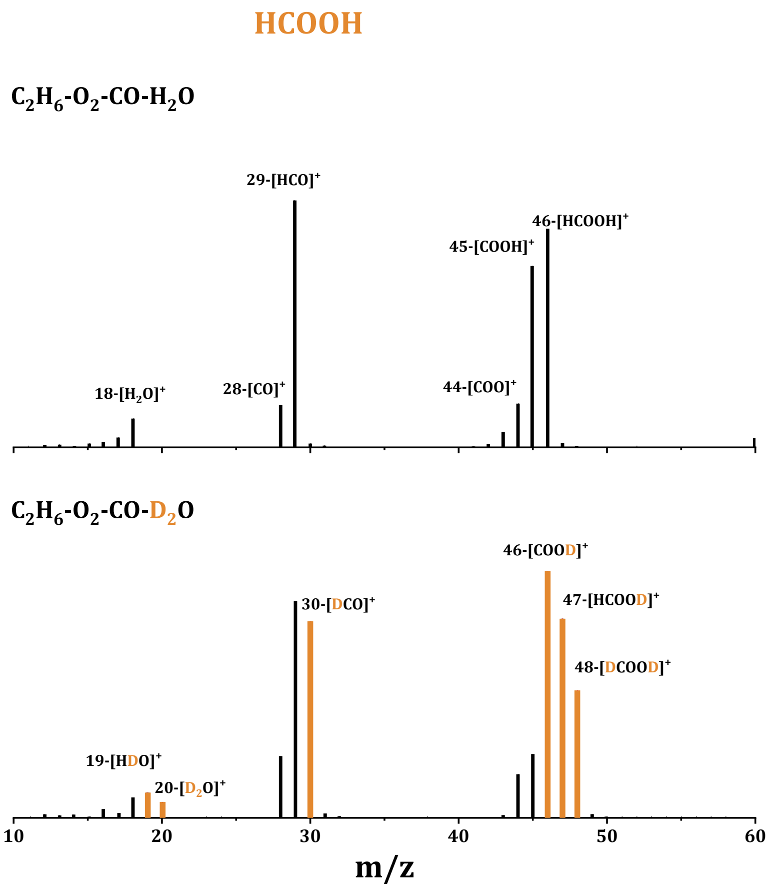

Figure S24. GC-MS analyses of formic acid during ^{18}O isotopic labeling experiments. Reaction conditions: 2.9 μmol Pd, 1.2 MPa O_2, 1 MPa CO, 2 MPa C_2H_6, 2 mL D_2O, 473 K, 2 h.

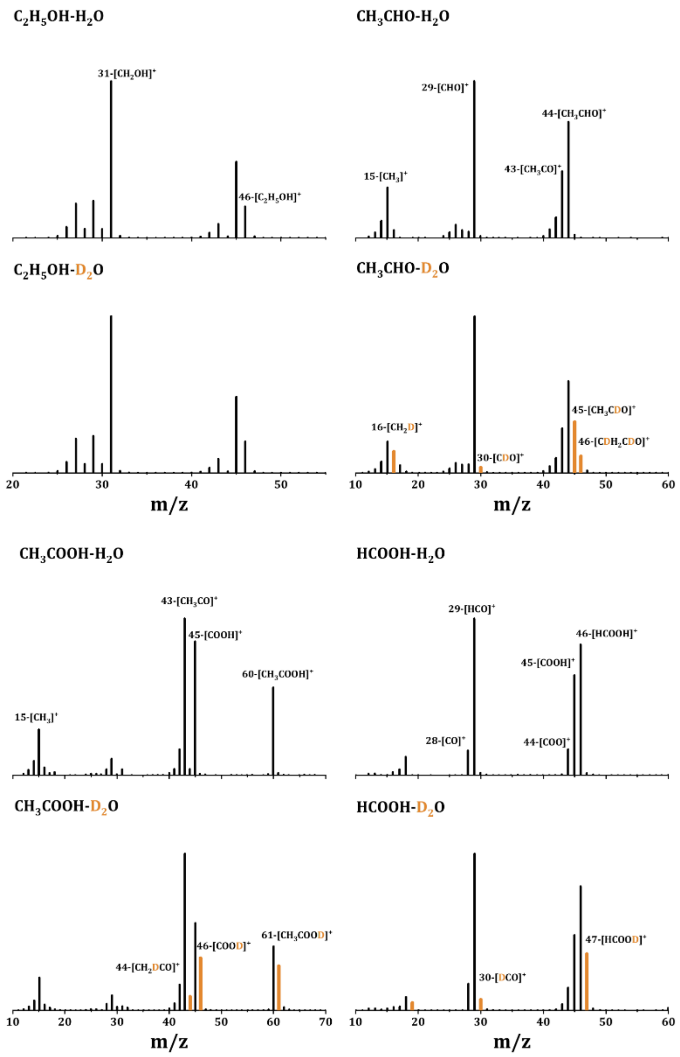

Figure S25. GC-MS analyses of C_2H_5OH, CH_3CHO, CH_3COOH and HCOOH under H-D exchange experiment. Reaction conditions: 2.9 μmol Pd, 1.5 mmol $C_2H_5OH/CH_3CHO/$ CH_3COOH/HCOOH, 2 mL D_2O, 4.2 MPa N_2, 473 K, 2 h.

Note: To further verify whether the H-D exchange occurs in liquid phase under the reaction condition, C_2H_5OH, CH_3CHO, CH_3COOH and HCOOH as well as D_2O were mixed at 473 K with the total pressure of N_2 over $PdCl_2$ catalyst. Typical D-labelled CH_3COOH, CH_3CHO and HCOOH fragments including CDH_2CO (*m/z*=44), COOD (*m/z*=46), CH_3COOD (*m/z*=61) and CH_2D (*m/z*=16), CDO (*m/z*=30), CH_3CDO (*m/z*=45), CH_2DCDO (*m/z*=46) and HDO (*m/z*=19), DCO (*m/z*=30), COOD (*m/z*=46), HCOOD (*m/z*=47) confirm the H-D exchange between CH_3COOH/CH_3CHO/HCOOH and D_2O which is not observed in the case of C_2H_5OH.

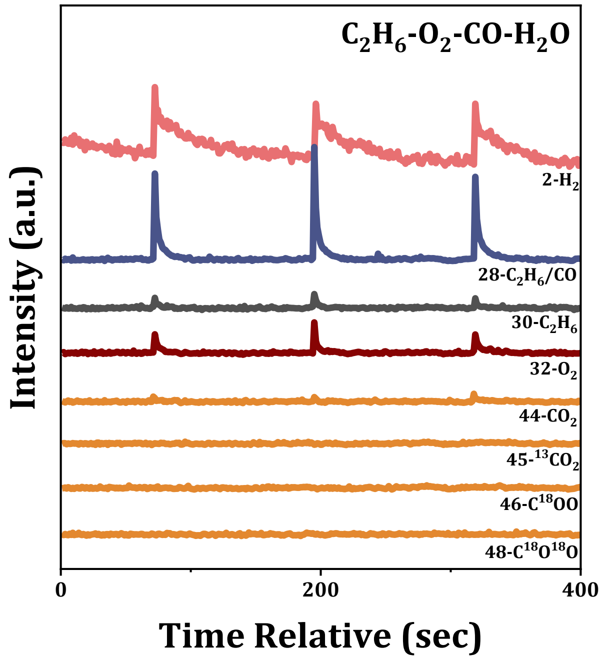

Figure S26. Mass spectrum of gas products during ethane oxidation. Reaction conditions: 2.9 μmol Pd, 1.2 MPa O_2, 1 MPa CO, 2 MPa C_2H_6, 10 mL H_2O, 473 K, 2 h.

Note: The typical signal of CO_2 (*m/z*=44) and H_2 (*m/z*=2) could be attributed to the water gas shift reaction ($CO+H_2O=CO_2+H_2$).

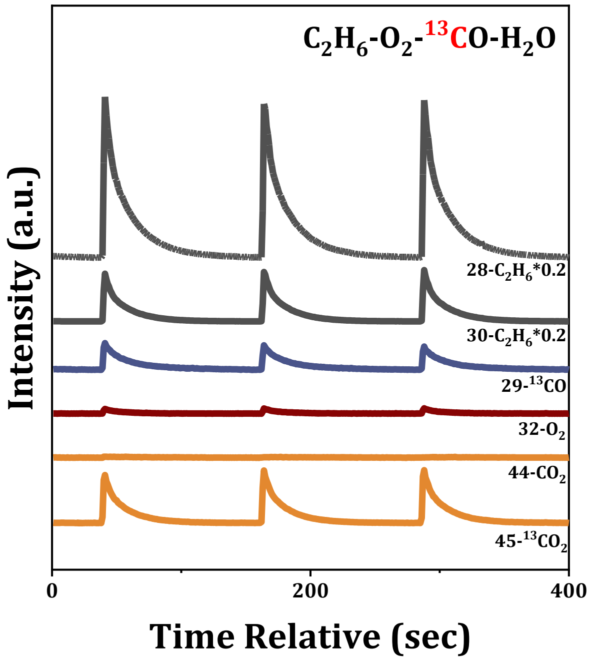

Figure S27. Mass spectrum of gas products during ethane oxidation with ^{13}CO. Reaction conditions: 2.9 μmol Pd, 0.3 MPa O_2, 0.25 MPa ^{13}CO, 0.5 MPa C_2H_6, N_2 was replenished to 4.2 MPa as a balance gas, 10 mL H_2O, 473 K, 2 h.

Note: The typical signal of $^{13}CO_2$ (*m/z*=45) could be attributed to the water gas shift reaction ($^{13}CO+H_2O=^{13}CO_2+H_2$).

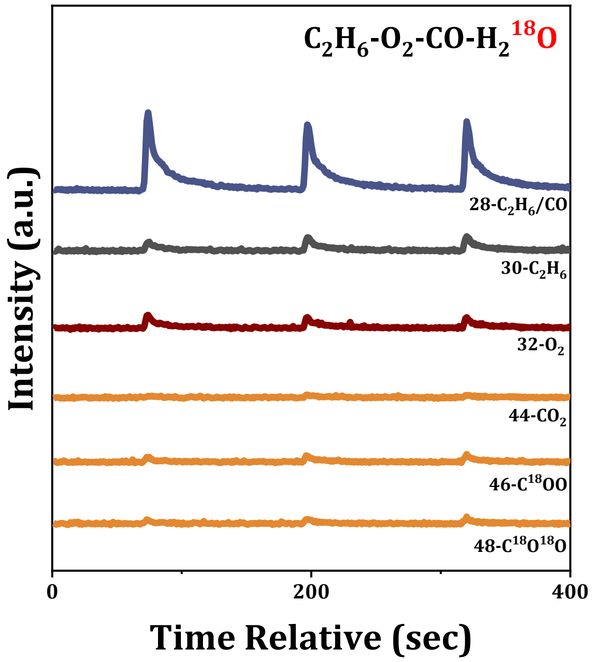

Figure S28. Mass spectrum of gas products during ethane oxidation with $H_2^{18}O$. Reaction conditions: 2.9 μmol Pd, 0.3 MPa O_2, 0.25 MPa $C^{18}O$, 0.5 MPa C_2H_6, 2 mL $H_2^{18}O$, 473 K, 2 h.

Note: The typical signal of $C^{18}OO$ (*m/z*=46) could be attributed to the water gas shift reaction ($CO+H_2^{18}O= CO^{18}O +H_2$).

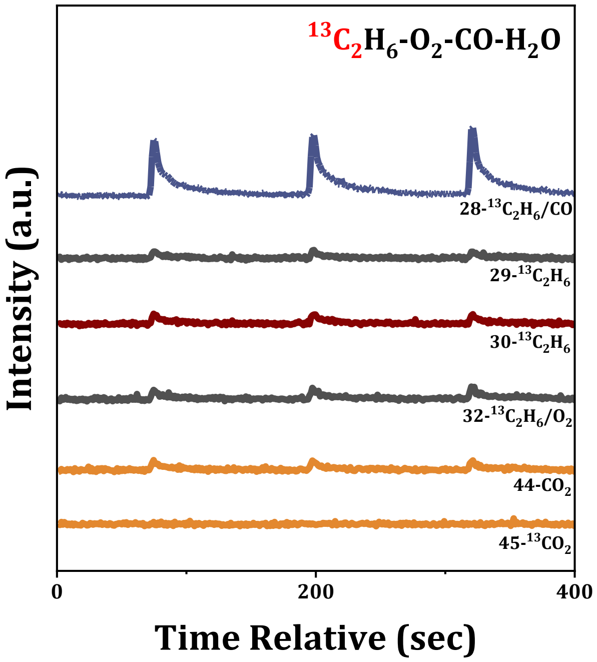

Figure S29. Mass spectrum of gas products during ethane oxidation with $^{13}C_2H_6$. Reaction conditions: 2.9 μmol Pd, 0.3 MPa O_2, 0.25 MPa CO, 0.1 MPa $^{13}C_2H_6$, 0.4 MPa C_2H_6, N_2 was replenished to 4.2 MPa as a balance gas, 10 mL H_2O, 473 K, 2 h.

Note: There is no signal of $^{13}CO_2$ (*m/z*=45), excluding the contribution of C_2H_6 towards CO_2.

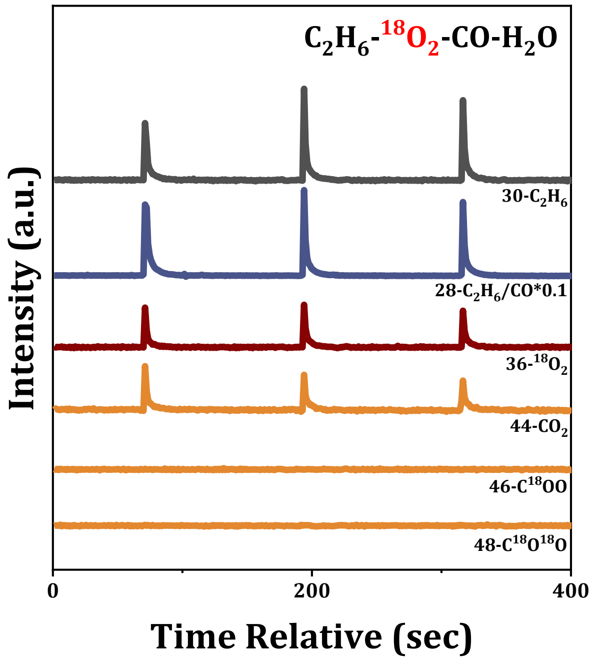

Figure S30. Mass spectrum of gas products during ethane oxidation with $^{18}O_2$. Reaction conditions: 2.9 μmol Pd, 0.3 MPa $^{18}O_2$, 0.25 MPa CO, 0.5 MPa C_2H_6, N_2 was replenished to 4.2 MPa as a balance gas, 10 mL H_2O, 473 K, 2 h.

Note: The typical signal of CO_2 (*m/z*=44) could be attributed to the water gas shift reaction ($CO+H_2O=CO_2+H_2$). Notably, none signal of $C^{18}O_2$ (*m/z*=46, 48) could be detected, further excluding contribution of O_2 towards CO_2.

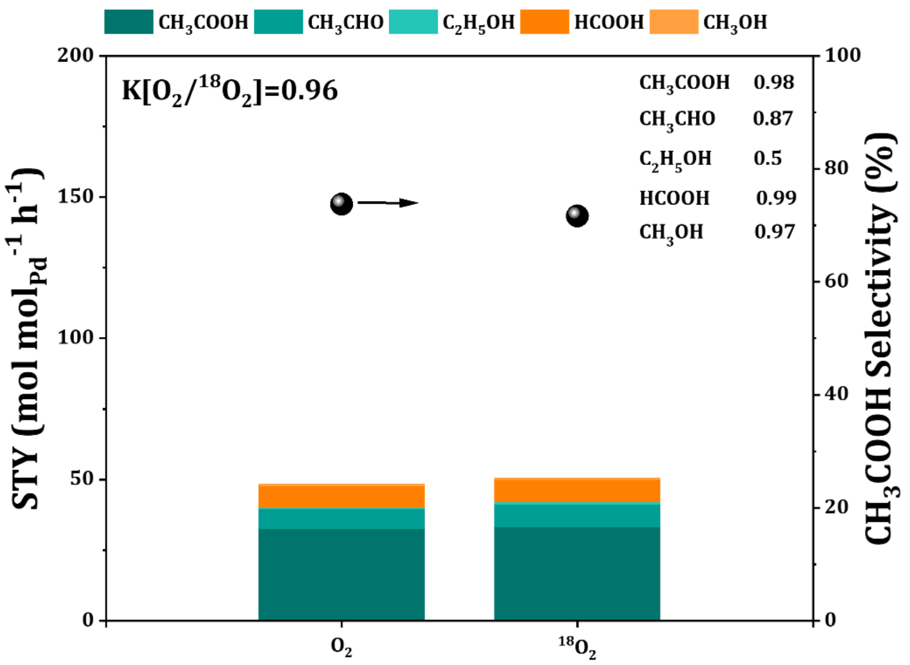

Figure S31. Kinetic isotope effects (KIE) of O_2/$^{18}O_2$ obtained from the product yield. Reaction conditions: 2.9 μmol Pd, 0.3 MPa O_2/$^{18}O_2$, 0.25 MPa CO, 0.5 MPa C_2H_6, 10 mL H_2O, 473 K, 2 h.

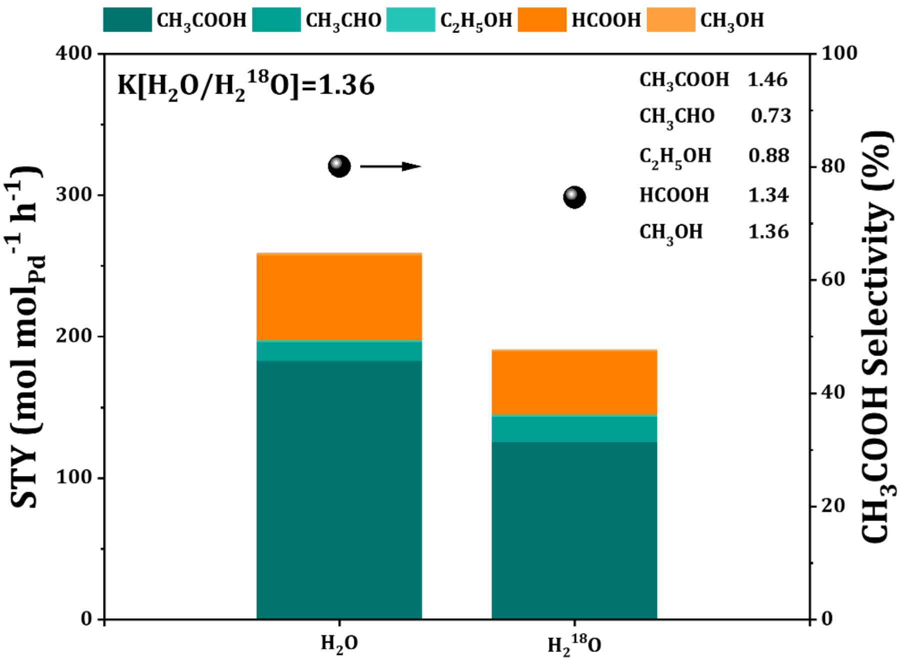

Figure S32. Kinetic isotope effects (KIE) of $H_2O/H_2{}^{18}O$ obtained from the product yield. Reaction conditions: 1.45 μmol Pd, 1.2 MPa O_2, 1 MPa CO, 2 MPa C_2H_6, 1 mL $H_2O/H_2{}^{18}O$, 473 K, 2 h

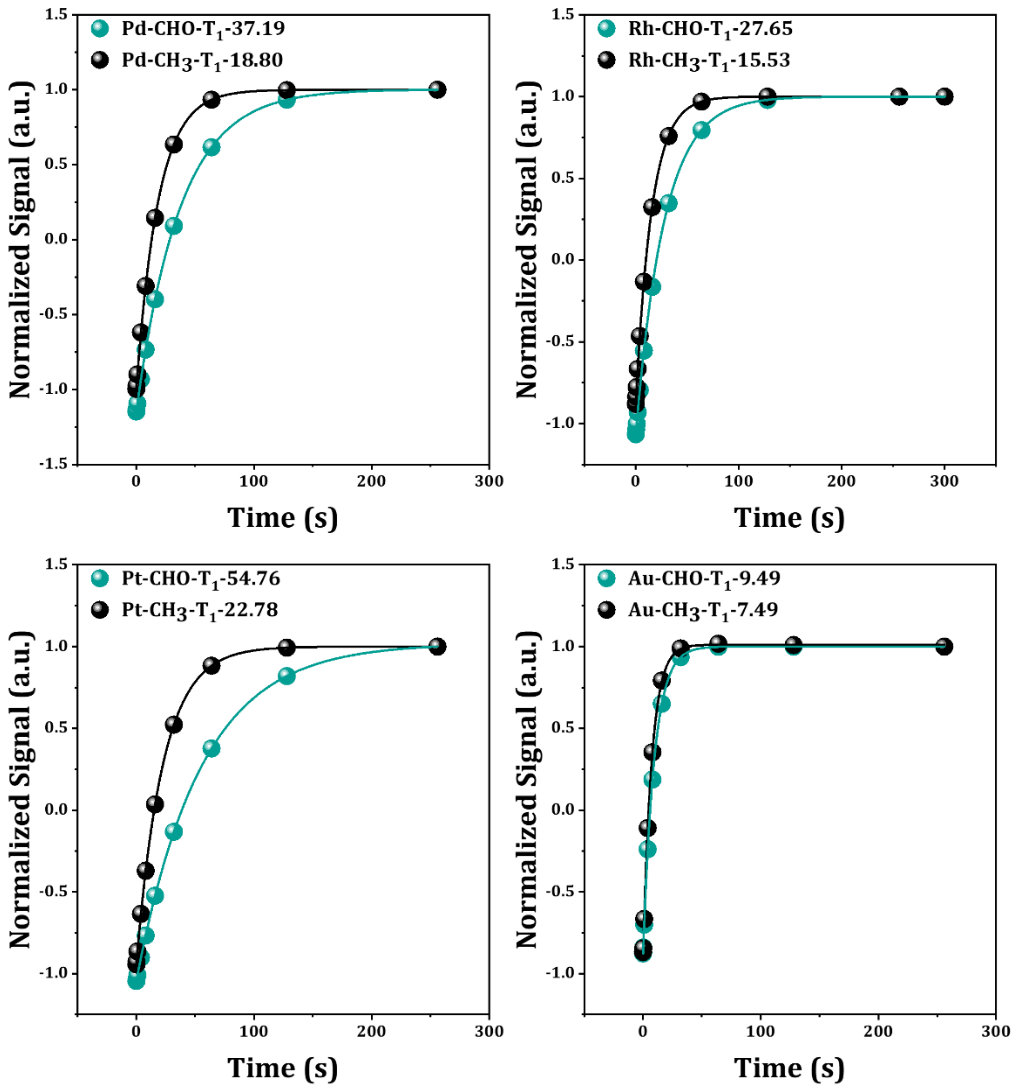

Figure S33. The relaxation time T_1 (longitudinal relaxation time) to acetaldehyde (CH_3 and CHO) for different catalysts measured by NMR.

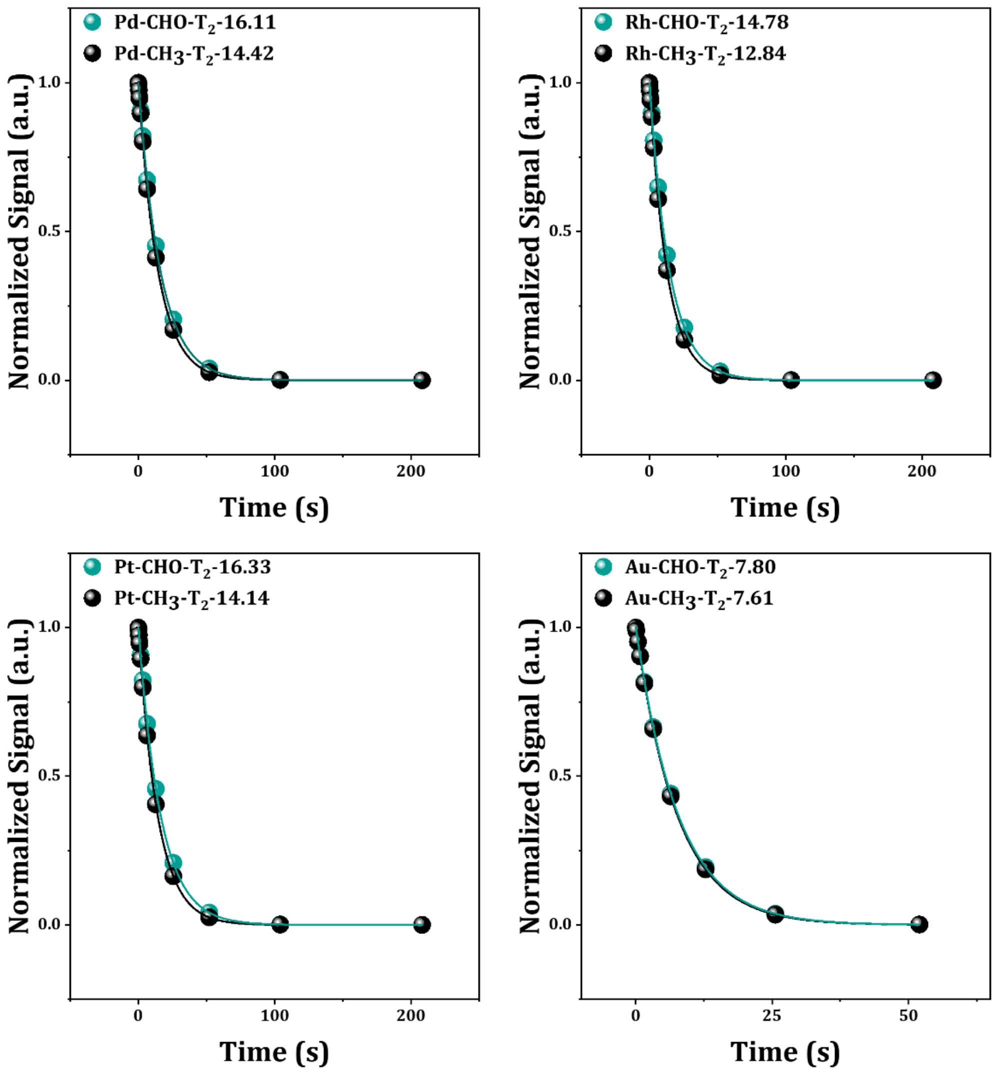

Figure S34. The relaxation time T_2 (longitudinal relaxation time) to acetaldehyde (CH_3 and CHO) for different catalysts measured by NMR.

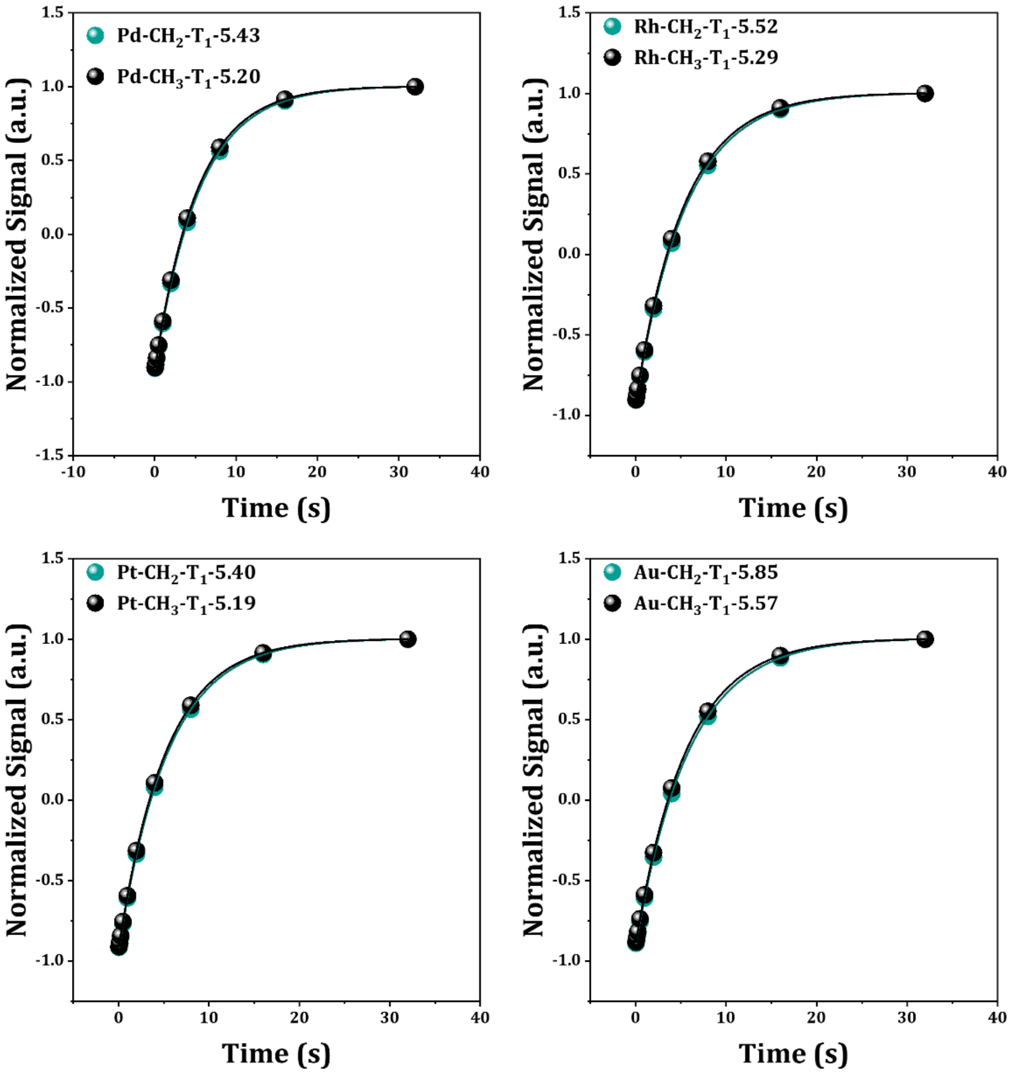

Figure S35. The relaxation time T_1 (longitudinal relaxation time) to ethanol (CH_3 and CH_2) for different catalysts measured by NMR.

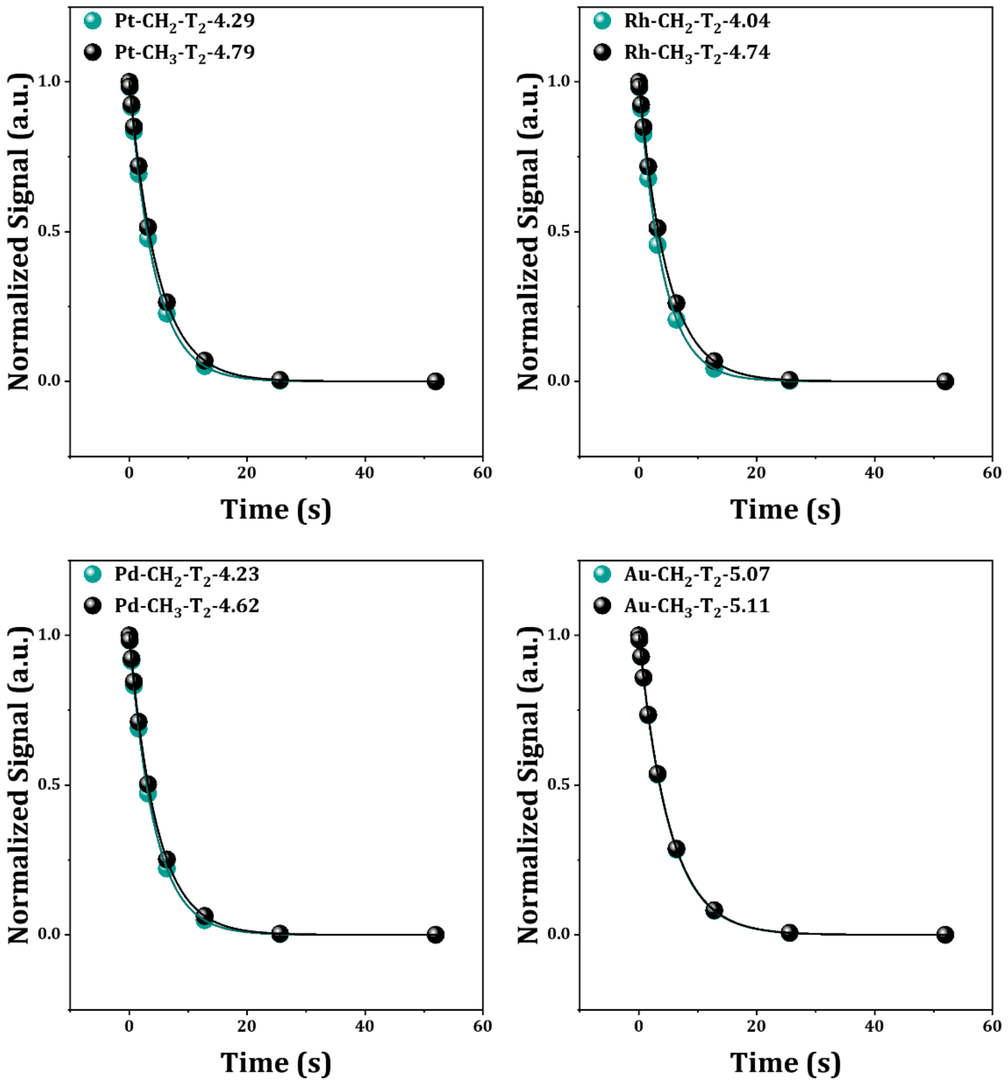

Figure S36. The relaxation time T_2 (longitudinal relaxation time) to ethanol (CH_3 and CH_2) for different catalysts measured by NMR.

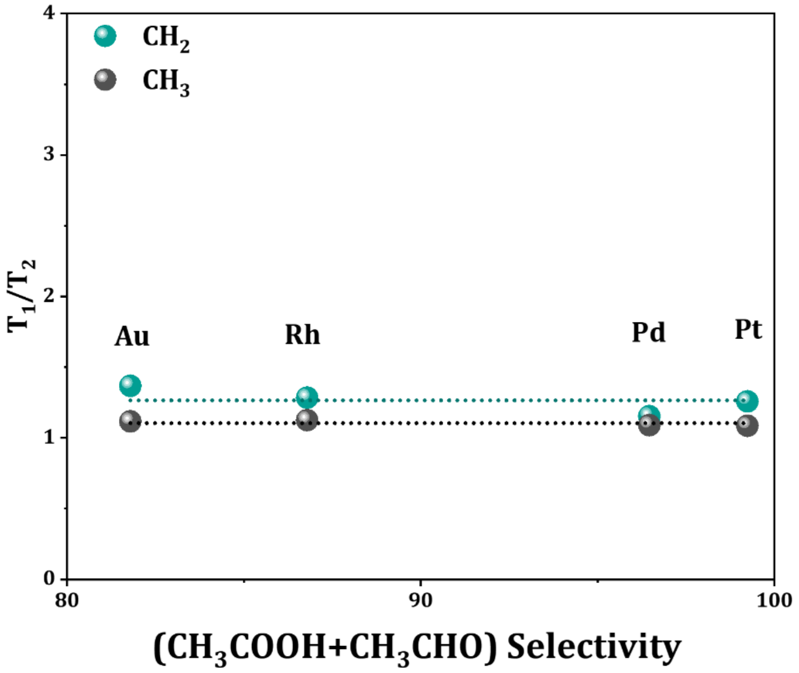

Figure S37. Acetate acid and acetaldehyde selectivity during ethane oxidation versus the T_1/T_2 ratio of ethanol measured by NMR.

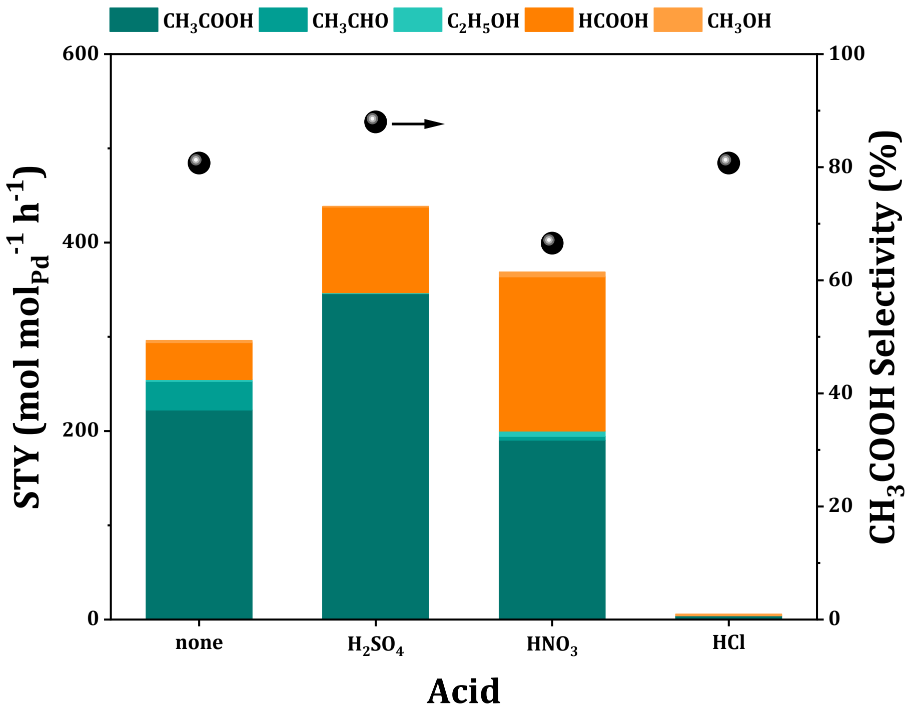

Figure S38. Catalytic performance of $PdCl_2$ with different acids in ethane oxidation. Reaction conditions: 2.9 μmol Pd, 1.2 MPa O_2, 1 MPa CO, 2 MPa C_2H_6, 10 mL H_2O, 0 or 0.2 mol L^{-1} H_2SO_4/HNO_3/HCl, 473 K, 2 h.

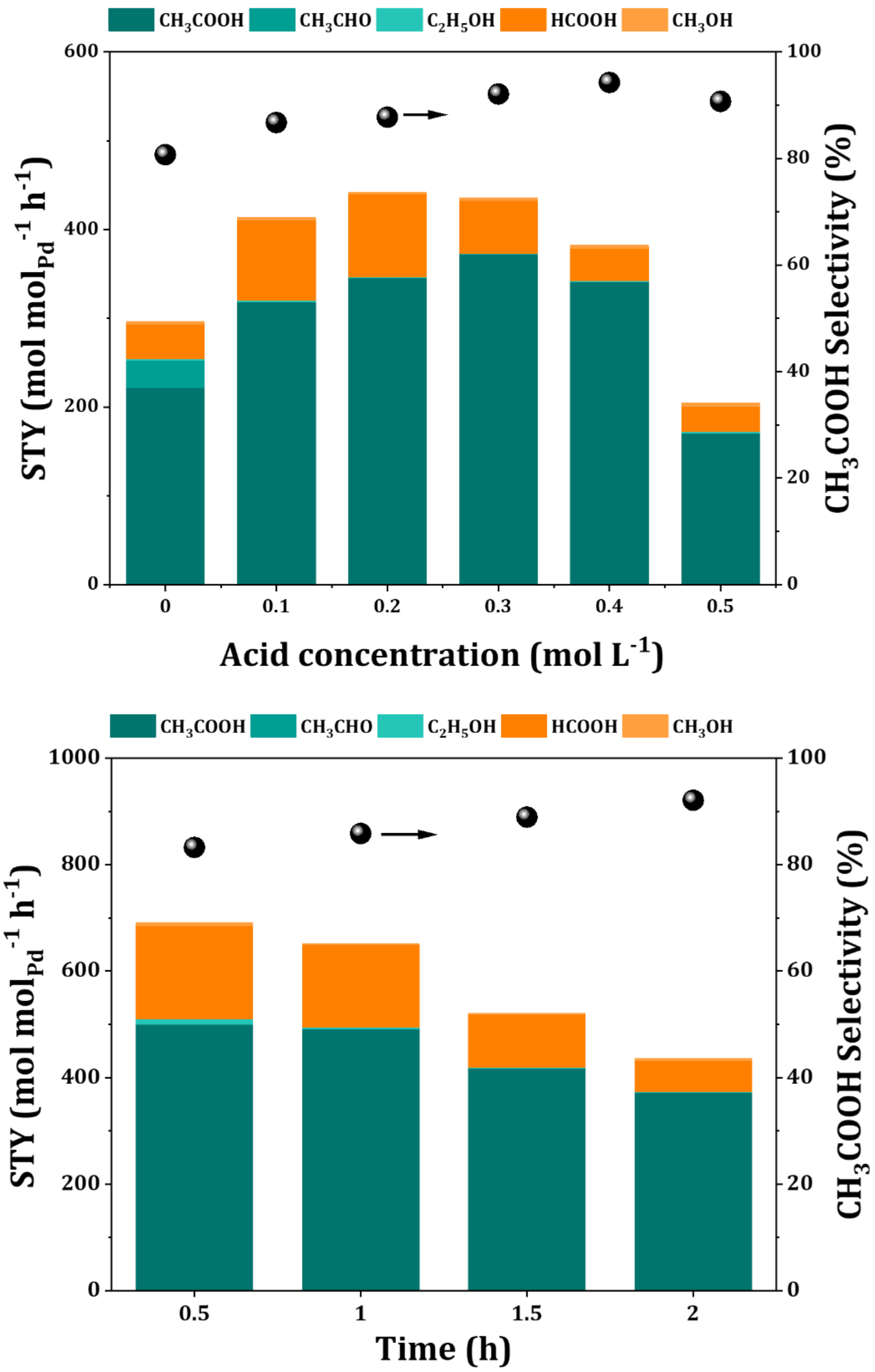

Figure S39. Catalytic performance of $PdCl_2$ with different concentrations of H_2SO_4 in ethane oxidation. Reaction conditions: 2.9 μmol Pd, 1.2 MPa O_2, 1 MPa CO, 2 MPa C_2H_6, 10 mL H_2O, 0-0.5 mol L^{-1} H_2SO_4, 473 K, 2 h; Time-dependent behaviors of ethane oxidation over $PdCl_2$ catalyst with H_2SO_4. Reaction conditions: 2.9 μmol Pd, 2.0 MPa C_2H_6, 1.2 MPa O_2, 1.0 MPa CO, 10 mL H_2O, 0.3 mol L^{-1} H_2SO_4, 473 K, 0.5-2 h.

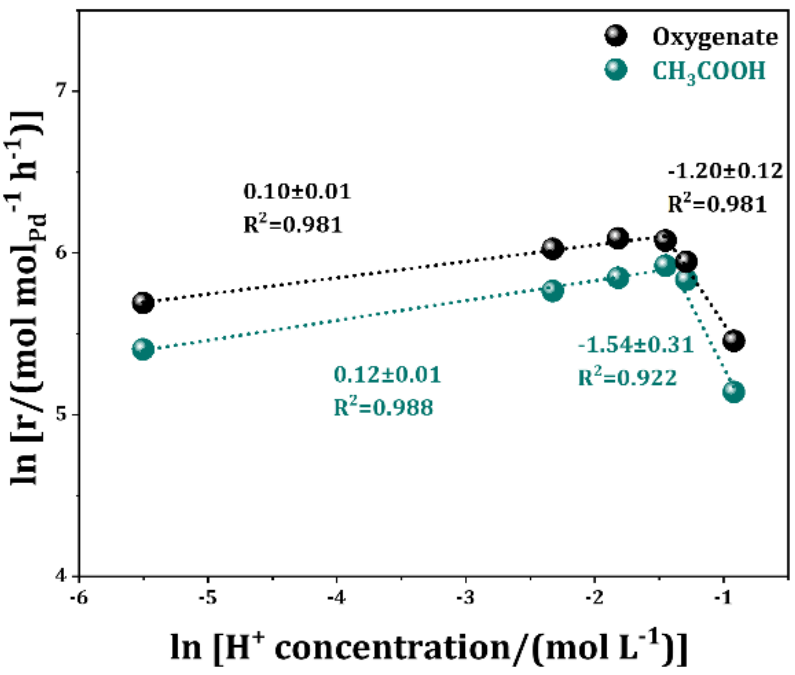

Figure S40. Relationship between H^+ concentration and the yield of liquid oxygenates as well as CH_3COOH. Reaction conditions: 2.9 μmol Pd, 1.2 MPa O_2, 1 MPa CO, 2 MPa C_2H_6, 10 mL H_2O, 473 K, 0-0.5 mol L^{-1} H_2SO_4, 2 h.

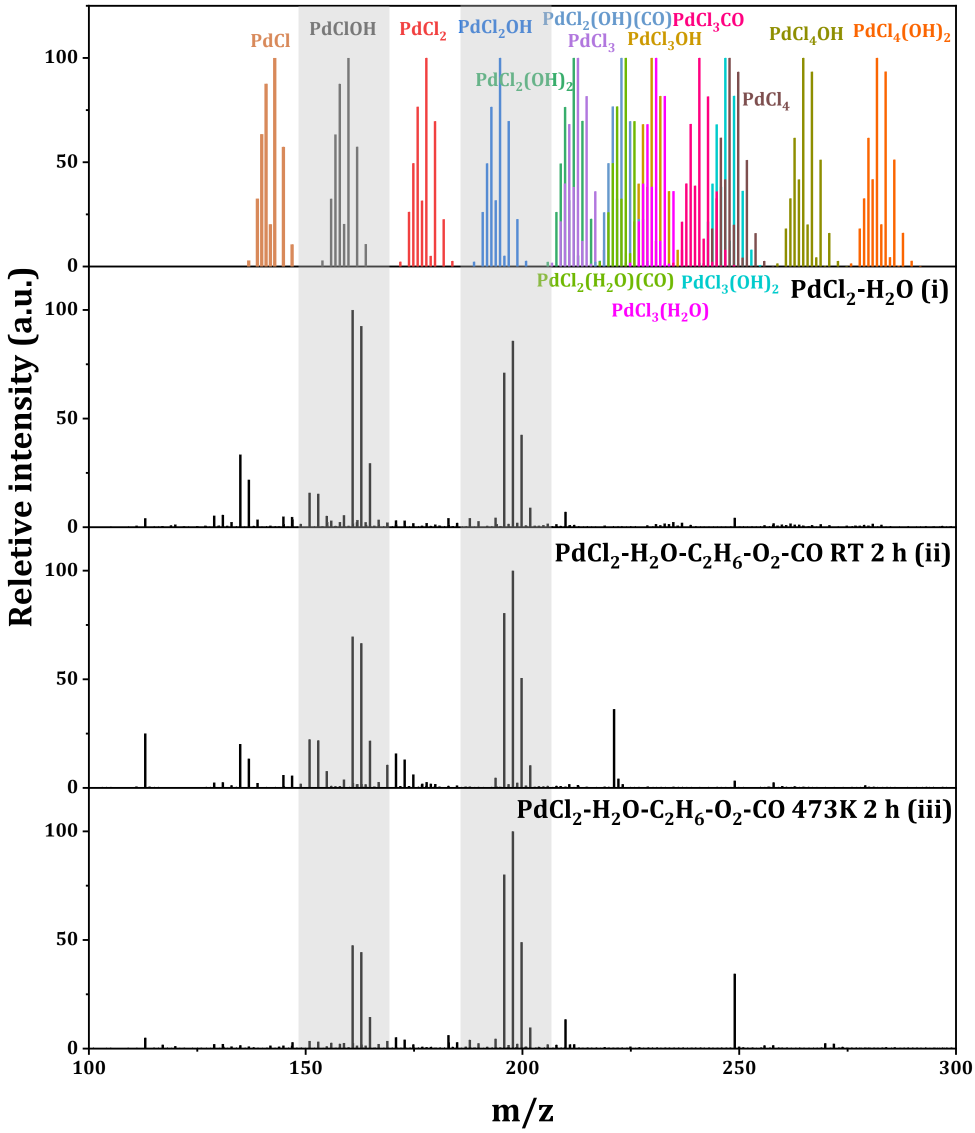

Figure S41. Mass spectrum of coordination situation of Pd under different conditions. Reaction conditions: (i) 2.9 μmol Pd, 10 mL H_2O; (ii) 2.9 μmol Pd, 1.2 MPa O_2, 1 MPa CO, 2 MPa C_2H_6, 10 mL H_2O, room temperature, 2 h; (iii) 2.9 μmol Pd, 1.2 MPa O_2, 1 MPa CO, 2 MPa C_2H_6, 10 mL H_2O, 473 K, 2 h.

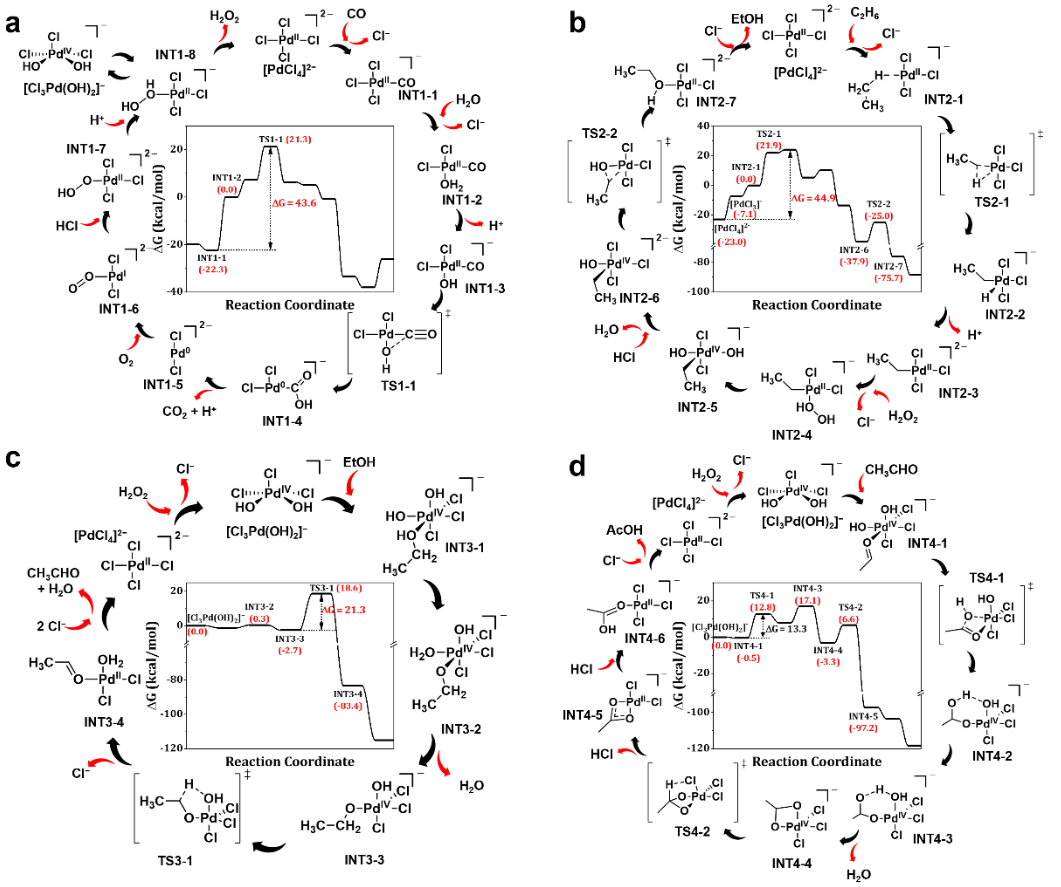

Figure S42. Reaction mechanism of ethane oxidation from DFT calculations. The Gibbs free energy profiles and reaction pathways of H_2O_2 generation (**a**), ethanol formation (**b**), acetaldehyde formation (**c**) and acetate acid formation (**d**). Oxidation states are explicitly labelled at the Pd center in all structures, except for all transition states and **INT2-2**. Within the standard definition of oxidation states, hydrogen in d-block metal complexes is typically assigned as hydride (H^-), and the Pd center in **INT2-2** would be assigned an oxidation state of +4.

**(A)**	**$PdCl_2$**	**$[PdCl_3]^-$**	**$[PdCl_4]^{2-}$**
Singlet	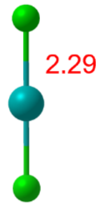	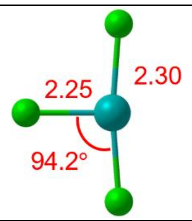	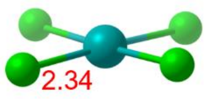
	ΔG = 79.0	ΔG = 15.9	ΔG = 0.0
Triplet	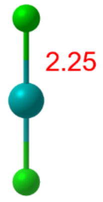	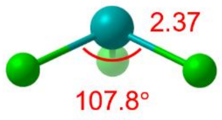	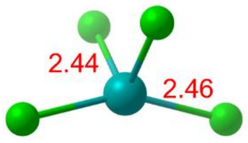
	ΔG = 63.6	ΔG = 32.2	ΔG = 31.7
Quintet	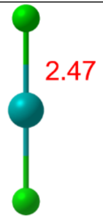	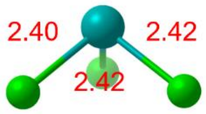	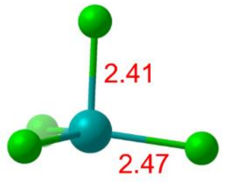
	ΔG = 58.7	ΔG = 140.1	ΔG = 140.7
**(B)**	**$Cl_2Pd(OH)_2$**	**$[Cl_3Pd(OH)_2]^-$**	**$[Cl_4Pd(OH)_2]^{2-}$**
Singlet	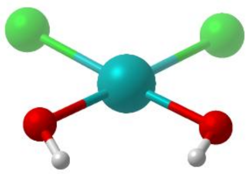	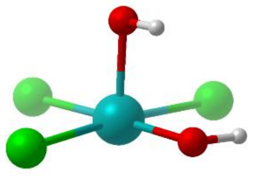	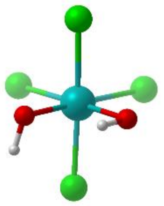
	ΔG = 39.7	ΔG = 14.9	ΔG = 0.0

Figure S43. The calculated relative Gibbs free energy (ΔG) for (A) $PdCl_2$, $[PdCl_3]^-$ and $[PdCl_4]^{2-}$ on singlet, triplet and quintet states, and (B) $Cl_2Pd(OH)_2$, $[Cl_3Pd(OH)_2]^-$ and $[Cl_4Pd(OH)_2]^{2-}$ on singlet states. (units: kcal/mol for energy and Å for bond length).

Note: The singlet $[PdCl_4]^{2-}$ has the lowest energy and should be the ground state of Pd(II) species. The stable state is singlet for $[PdCl_3]^-$ while quintet for $PdCl_2$. However, $^1[PdCl_3]^-$ and $^5PdCl_2$ are 15.9 and 58.7 kcal/mol higher in energy, respectively, and much less stable relative to $^1[PdCl_4]^{2-}$. Figure S41B shows that the singlet $[Cl_4Pd(OH)_2]^{2-}$ should be the stable form of Pd(IV) species, but it lacks of ability to cooperate substrate for the reaction. Therefore, $[Cl_3Pd(OH)_2]^-$ was chosen to be the active Pd(IV) species in reaction.

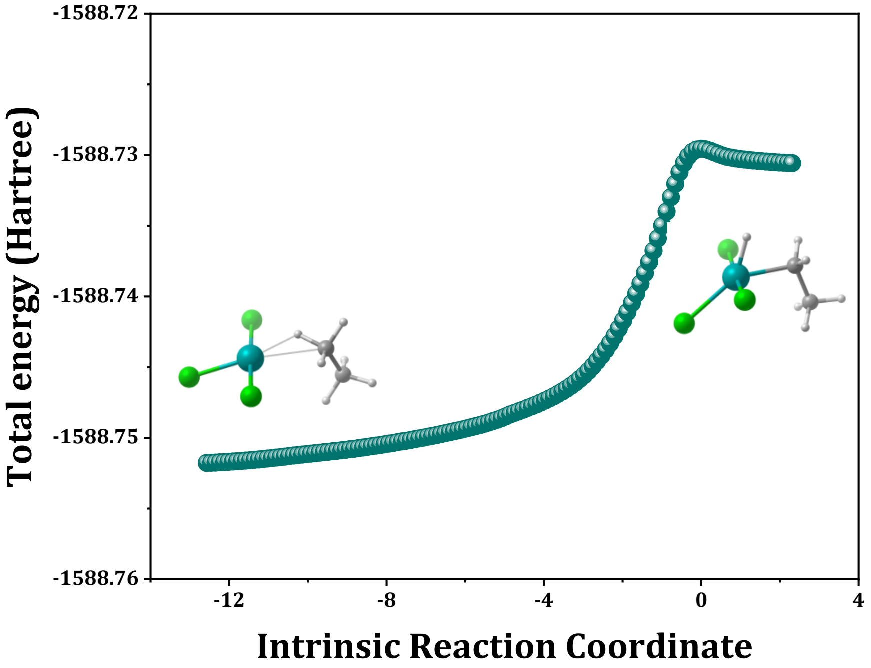

Figure S44. IRC calculation result of TS2-1. The structures of both sides are shown in **Figure S44**.

Table S1. Titration of H_2O_2 under various reaction conditions.

H_2O_2 amount (μmol)	Reaction conditions
7.4	2.9 μmol Pd, 10 mL H_2O, 473 K, 2 h, 2.0 MPa N_2, 1.2MPa O_2, 1.0 MPa CO
2.4	2.9 μmol Pd, 10 mL H_2O, 473 K, 2 h, 2 MPa C_2H_6, 1.2MPa O_2, 1 MPa CO
214.8	10 mL H_2O_2 (0.02 M)
25.6	2.9 μmol Pd, 10 mL H_2O_2 (0.02 M), 473 K, 2 h, 4.2 MPa N_2
0.5	2.9 μmol Pd, 10 mL H_2O_2 (0.02 M), 473 K, 2 h, 2 MPa C_2H_6, 2.2 MPa N_2

Table S2. Comparison of theoretical and actual conversion in ethane oxidation to CH_3COOH.

Partial pressure (MPa)	Theoretical maximum value (%)		Experimental
$C_6H_6/O_2/CO$	$H_2O_2/\cdot OH$ pathway	O_2 pathway	Conversion (%)
2.0/1.2/1.0	16.67	40	10.7
2.0/0.3/0.5	5	10	2.7
2.0/0.5/0.5	8.33	10.67	5.7
2.0/0.8/0.5	8.33	26.67	6.2
2.0/1.0/0.5	8.33	33.33	6.5
2.0/1.2/0.5	8.33	40	7.1
2.0/1.5/0.5	8.33	50	7.0
2.0/1.2/0.3	5	40	5.3
2.0/1.2/0.5	8.33	40	7.1
2.0/1.2/0.8	13.33	40	10.0
2.0/1.2/1.3	20	40	7.7
2.0/1.2/1.5	20	40	5.0
1.0/1.2/1.0	33.33	80	16.9
1.5/1.2/1.0	22.22	53.33	13.2
2.5/1.2/1.0	13.33	32	7.5
3.0/1.2/1.0	11.11	26.67	6.2

Note: O_2 pathway, $C_2H_6 + 1.5\ O_2 \rightarrow CH_3COOH + H_2O$

H_2O_2/ OH pathway, $C_2H_6 + 3\ O_2 + 3\ CO \rightarrow CH_3COOH + 3\ CO_2 + H_2O$

Table S3. ^{1}H NMR and ^{13}C NMR chemical shifts of ethane oxidation products[a].

Species	Formula	Chemical shift, multiplicity	Coupling constants for C-H ($^{1}J_{C\text{-}H}$)
Dimethyl sulfoxide (DMSO)	C**H**$_3$SOC**H**$_3$<br>**C**H$_3$SO**C**H$_3$	δ 2.64 ppm, s<br>δ 38.8 ppm, s	70 Hz
Water	H_2O	δ 4.70 ppm, s	--
Acetic acid	C**H**$_3$COOH<br>**C**H$_3$COOH	δ 2.00 ppm, s<br>δ 20.6 ppm, d	64 Hz
	CH$_3$**C**OOH	δ 176.8 ppm, d	--
Ethanol	C**H**$_3$CH$_3$OH<br>**C**H$_3$CH$_3$OH	δ 1.09 ppm, t<br>δ 16.9 ppm, d	--
	CH$_3$C**H**$_3$OH<br>CH$_3$**C**H$_3$OH	δ 3.57 ppm, q<br>δ 57.6 ppm, d	--
Acetaldehyde	C**H**$_3$CHO<br>**C**H$_3$CHO	δ 2.16 ppm, d<br>δ 30.2 ppm, d	64 Hz
	CH$_3$C**H**O<br>CH$_3$**C**HO	δ 9.59 ppm, q<br>δ 206.9 ppm, d	88 Hz
Acetaldehyde (hydrated)	C**H**$_3$CHO (hydrated)<br>**C**H$_3$CHO (hydrated)	δ 1.24 ppm, d<br>δ 23.3 ppm, d	64 Hz
	CH$_3$C**H**O (hydrated)<br>CH$_3$**C**HO (hydrated)	δ 5.16 ppm, q<br>δ 88.4 ppm, d	--
Propionic acid	C**H**$_3$CH$_2$COOH<br>CH$_3$C**H**$_2$COOH	δ 1.00 ppm, t<br>δ 2.30 ppm, q	--
Aceton	C**H**$_3$COCH$_3$	δ 2.07 ppm, s	--
Formic acid	**H**COOH<br>H**C**OOH	δ 8.15 ppm, s<br>δ 166.0 ppm, s	104 Hz
Methanol	C**H**$_3$OH<br>**C**H$_3$OH	δ 3.27 ppm, s<br>δ 49.1 ppm, s	72 Hz

[a]: s=singlet, d=doublet, t=triplet, q=quartet, br=broad, m=multiplet.

Table S4. Performance of different catalyst systems in ethane oxidation.

Catalyst	Reaction Condition	Oxygenate Productivity ($mol/mol_{metal}/h$)							Conv. (%)	Ref.
		**CH_3COOH**	**CH_3CHO**	**C_2H_5OH**	**HCOOH**	**CH_3OH**	**C_2H_5COOH**	**C_2H_4**		
Rh_1/AC	3 MPa C_2H_6, 0.5 MPa O_2, 1 MPa CO<br>10 mL H_2O, 423 K, 2 h	11.7	46.8	7.5	0	0	0	0	0.6	22
Ir_n/ND	1.05 MPa C_2H_6, 0.075 MPa O_2, 0.24 MPa CO, 1.635 MPa noble gas<br>15 mL H_2O, 373 K, 3 h	2.3	3.6	1.2	0	0.08	0.4	0.12	0.005	8
Pd/ZSM-5	2 MPa C_2H_6, 0.4 MPa O_2, 0.5 MPa CO<br>10 mL H_2O, 423 K, 1 h	7.0	13.2	1.7	4.4	0.5	0	0	0.007	39
Pd/C+$CuCl_2$	2.758 MPa C_2H_6, 0.517 MPa O_2, 1.034 MPa CO, 2.758 MPa N_2<br>1 mL H_2O, 3 mL CF_3COOH, 0.1 mmol $CuCl_2$, 363 K, 16 h	18.6	0	99.8	0	39.9	0	0	0.03	25
Pd/C	3.447 MPa C_2H_6, 0.689 MPa O_2, 0.689 MPa CO<br>5 mL 0.1 M DCl in D_2O, 373 K, 22 h	51.7	0	0	4.6	0	0	0	0.02	5
Pd_1/g-C_3N_4 light	10 mL/min, 10% C_2H_6/Ar, 30 mL H_2O,1.5 mmol H_2O_2, 0.02 mmol H_2SO_4, 0.05 mmol $FeSO_4$, 273 K, 1 h	254.6	0	0	11.4	0	0	0	1.8	3
$Nb_{0.6}PMo_{12}$ Pyr	Flow: Ethane: oxygen: helium: steam =16:8:16:20 mL/min, GHSV=4500 h^{-1}, 653 K	1.18	0	0	0	0	0	7.68	6	40
$Pd/Mo_{0.61}V_{0.31}Nb_{0.08}O_x/TiO_2$	0.533 MPa C_2H_6, 0.107 MPa O_2, 0.32 MPa H_2O, 0.64 MPa noble gas, 573 K	13.8[a]	0	0	0	0	0	0.1[a]	5.1	41
$[VO_2\{HB(3,5\text{-}Me_2pz)_3\}]$	0.5 MPa C_2H_6, 0.5 MPa CO<br>4 mmol $K_2S_2O_8$, 5.5 mL CF_3COOH, 353 K, 20 h	0.5	0	0	0	0	0.6	0	35.3	42
$[VO_2\{HC(pz)_3\}][BF_4]$	0.5 MPa C_2H_6, 0.5 MPa CO<br>4 mmol $K_2S_2O_8$, 5.5 mL CF_3COOH, 353 K, 20 h	0.5	0	0	0	0	0.5	0	31.4	42
$Ca[V\{ON[CH(CH_3)COO]_2\}_2]$	1 MPa C_2H_6, 3 MPa CO, 4.00 mmol $K_2S_2O_8$,<br>4 mL CF_3COOH, 353 K, 20 h	98.1	0	0	0	0	667.9	0	19.5	43
	2 MPa C_2H_6, 1.2 MPa O_2, 1 MPa CO<br>10 mL H_2O, 473 K, 0.5 h	435.2	74.1	13.8	92.3	9.9	0	0	5.6	
$PdCl_2$	2 MPa C_2H_6, 1.2 MPa O_2, 1 MPa CO<br>10 mL H_2O, 473 K, 2 h	222.3	30.4	2.0	39.1	2.3	0	0	10.7	This Work
	2 MPa C_2H_6, 1.2 MPa O_2, 1 MPa CO<br>10 mL H_2O, 0.3 mol L^{-1} H_2SO_4, 473 K, 2 h	372.7	0	0.9	59.5	2.6	0	0	15.7	

[a] The unit is mol kg_{cat}^{-1} h^{-1}

Table S5. MN15-D3(BJ)/ma-def2-TZVPP/SMD(water)//TPSS-D3(BJ)/ma-def2-TZVP/IEFPCM (water) computed electronic energy, thermal energies, enthalpies, free energies of all stationary points at 473.15 K.

Species	E_0	E_0+ZPE	U	H	G
**$^{3}O_2$**	-150.235322	-150.231789	-150.227981	-150.226483	-150.265927
**CO**	-113.235037	-113.230171	-113.226411	-113.224913	-113.262945
**CO_2**	-188.473143	-188.461885	-188.457058	-188.455560	-188.498553
**HCl**	-460.722857	-460.716273	-460.712526	-460.711028	-460.747075
**Cl^-**	-460.287974	-460.287974	-460.285727	-460.284229	-460.313535
**H_2O**	-76.398745	-76.378021	-76.373471	-76.371973	-76.409869
**H_3O^+**	-76.816183	-76.781719	-76.776897	-76.775399	-76.814937
**H_2O_2**	-151.482632	-151.457087	-151.451274	-151.449776	-151.495754
**C_2H_6**	-79.737485	-79.664015	-79.656913	-79.655415	-79.704274
**EtOH**	-154.928071	-154.849596	-154.840481	-154.838983	-154.894308
**CH_3CHO**	-153.718911	-153.664407	-153.656677	-153.655179	-153.707844
**AcOH**	-228.948376	-228.888185	-228.878558	-228.877060	-228.936571
**$^{1}PdCl_2$**	-1047.890418	-1047.888666	-1047.880461	-1047.878963	-1047.937115
**$^{3}PdCl_2$**	-1047.918335	-1047.916947	-1047.911380	-1047.909882	-1047.961511
**$^{5}PdCl_2$**	-1047.920823	-1047.919536	-1047.910987	-1047.909489	-1047.969446
**$^{1}[PdCl_3]^-$**	-1508.297224	-1508.294286	-1508.283355	-1508.281857	-1508.351095
**$^{3}[PdCl_3]^-$**	-1508.264966	-1508.262713	-1508.251228	-1508.249730	-1508.325231
**$^{5}[PdCl_3]^-$**	-1508.093822	-1508.091534	-1508.080118	-1508.078620	-1508.153230
**$^{1}[PdCl_4]^{2-}$**	-1968.630307	-1968.626331	-1968.611878	-1968.610380	-1968.690004
**$^{3}[PdCl_4]^{2-}$**	-1968.574196	-1968.571112	-1968.555933	-1968.554435	-1968.639535
**$^{5}[PdCl_4]^{2-}$**	-1968.396417	-1968.393770	-1968.378209	-1968.376711	-1968.465810
**$Cl_2Pd(OH)_2$**	-1199.439904	-1199.411904	-1199.396403	-1199.394905	-1199.473727
**$[Cl_3Pd(OH)_2]^-$**	-1659.78981	-1659.760192	-1659.741468	-1659.73997	-1659.826633
**$[Cl_4Pd(OH)_2]^{2-}$**	-2120.12369	-2120.093104	-2120.070888	-2120.06939	-2120.163991
**INT1-1**	-1621.589527	-1621.578561	-1621.562749	-1621.561251	-1621.643409
**INT1-2**	-1237.674505	-1237.639183	-1237.621765	-1237.620267	-1237.704128
**INT1-3**	-1237.217219	-1237.194487	-1237.177876	-1237.176378	-1237.258981
**INT1-4**	-1237.220478	-1237.195933	-1237.180374	-1237.178876	-1237.260602
**INT1-5**	-1048.291344	-1048.289856	-1048.282169	-1048.280671	-1048.330264
**INT1-6**	-1198.536799	-1198.531351	-1198.517456	-1198.515958	-1198.594619
**INT1-6**	-1198.544709	-1198.539417	-1198.525311	-1198.523813	-1198.605928
**INT1-7**	-1659.357668	-1659.338535	-1659.321105	-1659.319607	-1659.404749
**INT1-8**	-1659.809818	-1659.778657	-1659.760528	-1659.75903	-1659.845673
**INT2-1**	-1588.051518	-1587.973253	-1587.952782	-1587.951284	-1588.044094
**INT2-2**	-1588.01185	-1587.936221	-1587.915978	-1587.91448	-1588.005713
**INT2-2**	-2048.328209	-2048.255526	-2048.230966	-2048.229468	-2048.334843
**INT2-3**	-1587.598987	-1587.532642	-1587.5131	-1587.511602	-1587.60207
**INT2-4**	-1278.788782	-1278.696215	-1278.67185	-1278.670352	-1278.776238
**INT2-5**	-1278.835754	-1278.743163	-1278.719295	-1278.717797	-1278.814441
**INT2-6**	-1663.199174	-1663.118323	-1663.095196	-1663.093699	-1663.190423
**INT2-7**	-1663.260784	-1663.176642	-1663.154325	-1663.152827	-1663.25061
**INT3-1**	-1814.75073	-1814.640132	-1814.60978	-1814.608282	-1814.72306
**INT3-2**	-1814.749662	-1814.639474	-1814.609316	-1814.607818	-1814.720447
**INT3-3**	-1738.323311	-1738.23834	-1738.213058	-1738.211561	-1738.315378
**INT3-4**	-1278.141161	-1278.057123	-1278.034046	-1278.032548	-1278.130522
**INT4-1**	-1813.543639	-1813.456651	-1813.42791	-1813.426412	-1813.535294

**INT4-2**	-1813.533709	-1813.4444	-1813.41731	-1813.415812	-1813.521694
**INT4-3**	-1813.513249	-1813.428177	-1813.400859	-1813.399361	-1813.507271
**INT4-4**	-1352.719892	-1352.647161	-1352.623201	-1352.621703	-1352.723288
**INT4-5**	-1276.516626	-1276.464311	-1276.445824	-1276.444327	-1276.532382
**INT4-6**	-1737.277753	-1737.212431	-1737.189142	-1737.187645	-1737.28953
**TS1-1**	-1237.196971	-1237.17451	-1237.159452	-1237.157954	-1237.236699
**TS2-1**	-1588.015593	-1587.941132	-1587.921526	-1587.920028	-1588.009153
**TS2-2**	-1663.179601	-1663.098868	-1663.076942	-1663.075444	-1663.169759
**TS3-1**	-1738.286748	-1738.207195	-1738.182704	-1738.181206	-1738.281356
**TS4-1**	-1813.527087	-1813.439296	-1813.412684	-1813.411186	-1813.51411
**TS4-2**	-1737.101788	-1737.043559	-1737.021642	-1737.020144	-1737.1141

Note**:** The electronic energy calculated using MN15-D3(BJ)/ma-def2-TZVPP/SMD (water) method based on geometries obtained by TPSS-D3(BJ)/ma-def2-TZVP/IEFPCM (water) method. U, H, G are the thermal energy, enthalpy and Gibbs free energy obtained by adding the thermal corrections obtain from TPSS-D3(BJ)/ma-def2-TZVP/IEFPCM (water) method to E_0. The unit of all the energies in **Table S5** is Hartree.

References

30. Frisch MJ, Trucks GW, Schlegel HB *et al*. *Gaussian 16 Revision A.03*. Wallingford: Gaussian Inc, 2016.
31. Tao JM, Perdew JP, Staroverov VN *et al*. Climbing the density functional ladder: Nonempirical meta-generalized gradient approximation designed for molecules and solids. *Phys Rev Lett* 2003; **91**: 146401.
32. Weigend F and Ahlrichs R. Balanced basis sets of split valence triple zeta valence and quadruple zeta valence quality for H to Rn: Design and assessment of accuracy. *Phys Chem Chem Phys* 2005; **7**: 3297-305.
33. Zheng J, Xu X, Truhlar DG. Minimally augmented Karlsruhe basis sets. *Theor Chem Acc* 2011; **128**: 295-305.
34. Cancès E, Mennucci B, Tomasi J. A new integral equation formalism for the polarizable continuum model: Theoretical background and applications to isotropic and anisotropic dielectrics. *J Chem Phys* 1997; **107**: 3032-41.
35. Yu H, He X, Li S *et al*. MN15: A Kohn-Sham global-hybrid exchange-correlation density functional with broad accuracy for multi-reference and single-reference systems and noncovalent interactions. *Chem Sci* 2016; **7**: 5032-51.
36. Marenich AV, Cramer CJ, Truhlar DG. Universal solvation model based on solute electron density and on a continuum model of the solvent defined by the bulk dielectric constant and atomic surface tensions. *J Phys Chem B* 2009; **113**: 6378-96.
37. Grimme S, Ehrlich S, Goerigk L. Effect of the damping function in dispersion corrected density functional theory. *J Comput Chem* 2011; **32**: 1456-65.
38. Kenichi F. The path of chemical reactions - The IRC approach. *J Am Chem Soc* 1981; **14**: 363-8.
39. Ye J, Zhang S, Guo Y et al. Enabling direct oxidation of ethane to acetaldehyde with oxygen using supported PdO nanoparticles. *Chem Commun* 2024; **60**: 6067-70.
40. Lin M and Sen A. A highly catalytic system for the direct oxidation of lower alkanes by dioxygen in aqueous medium: A formal heterogeneous analog of alkane monooxygenases. *J Am Chem Soc* 1992; **114**: 7307-8.
41. Li X and Iglesia E. Support and promoter effects in the selective oxidation of ethane to acetic acid catalyzed by Mo-V-Nb oxides. *Appl Catal A-Gen* 2008; **334**: 339-47.
42. Silva TFS, Luzyanin KV, Kirillova MV *et al*. Novel scorpionate and pyrazole dioxovanadium complexes catalysts for carboxylation and peroxidative oxidation of alkanes. *Adv Synth Catal* 2010; **352**: 171-87.
43. Kirillova MV, Kuznetsov ML, Da Silva JAL *et al*. Amavadin and other vanadium complexes as remarkably efficient catalysts for one-pot conversion of ethane to propionic and acetic acids. *Chem Eur J* 2008; **14**: 1828-42.
